# Correction: Impaired hepatic mitochondrial function during early lactation in dairy cows: Association with protein lysine acetylation

**DOI:** 10.1371/journal.pone.0215395

**Published:** 2019-04-10

**Authors:** The PLOS ONE Staff

## Notice of republication

This article was republished on March 27, 2019 to correct for significant typographical errors introduced during the typesetting process. The publisher apologizes for the errors. Please download this article again to view the correct version. The originally published, uncorrected article and the republished, corrected article are provided here for reference.

## Supporting information

S1 File Originally published, uncorrected article. (PDF)

S2 File Republished, corrected article. (PDF)
