# Supplementary figures and images for: Impaired hepatic mitochondrial function during early lactation in dairy cows: Association with protein lysine acetylation
Source: PLoS One. 2019 Mar 14;14(3):e0213780. doi: 10.1371/journal.pone.0213780 (PMC6417696; doi:10.1371/journal.pone.0213780)

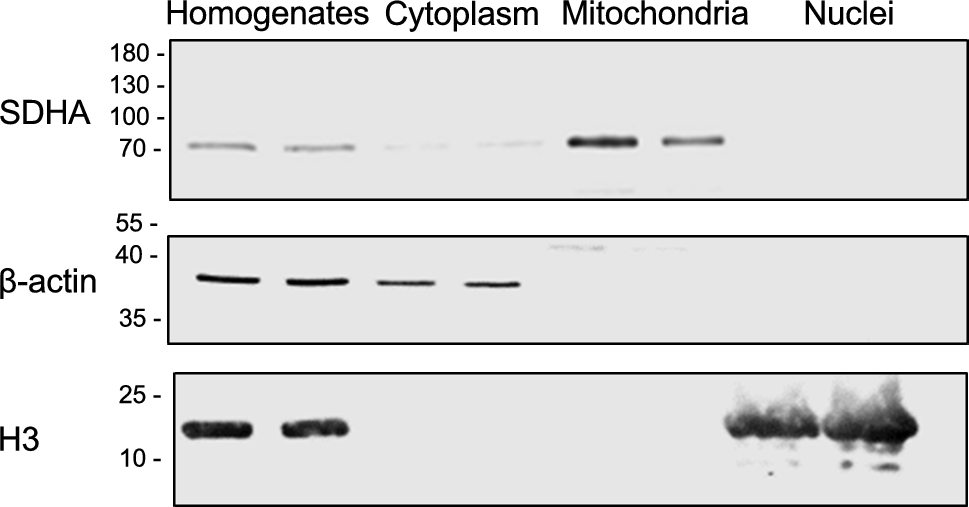

Supplement: S1 Fig — Liver biopsies were homogenized and subcellular fractions enriched in mitochondria, nuclei and cytosol were obtained as described previously [33]. Proteins from the different fractions were resolved by SDS/PAGE and Western blots performed with antibodies against proteins from mitochondria (SDHA), cytosol (β-actin) and nuclei (histone H3). (TIF) [file pone.0213780.s001.tif]

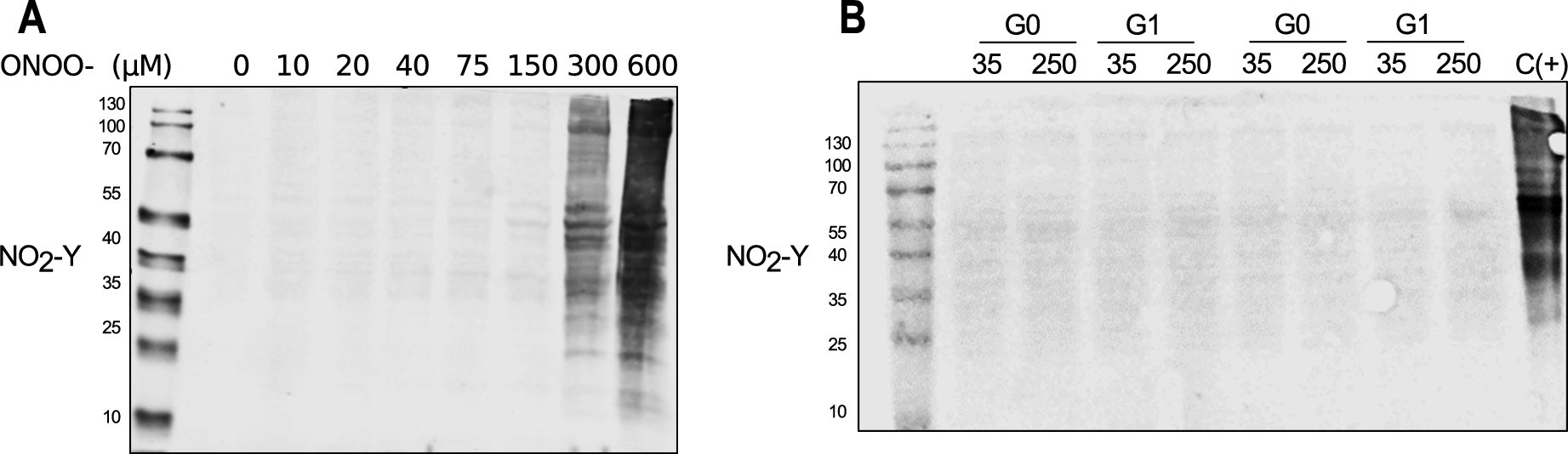

Supplement: S2 Fig — (A) Representative western blot of liver homogenates exposed to different concentrations of peroxynitrite (ONOO-) in 100 mM phosphate buffer pH 7.4. (B) Representative western blot of 3-nitrotyrosine in liver homogenates of G0 and G1 cows at 35 and 250 DPP, and a positive control (C (+)). The positive control was obtained exposing the bovine serum albumin to 300 μM peroxynitrite in 100 mM phosphate buffer pH 7.4. (TIF) [file pone.0213780.s002.tif]

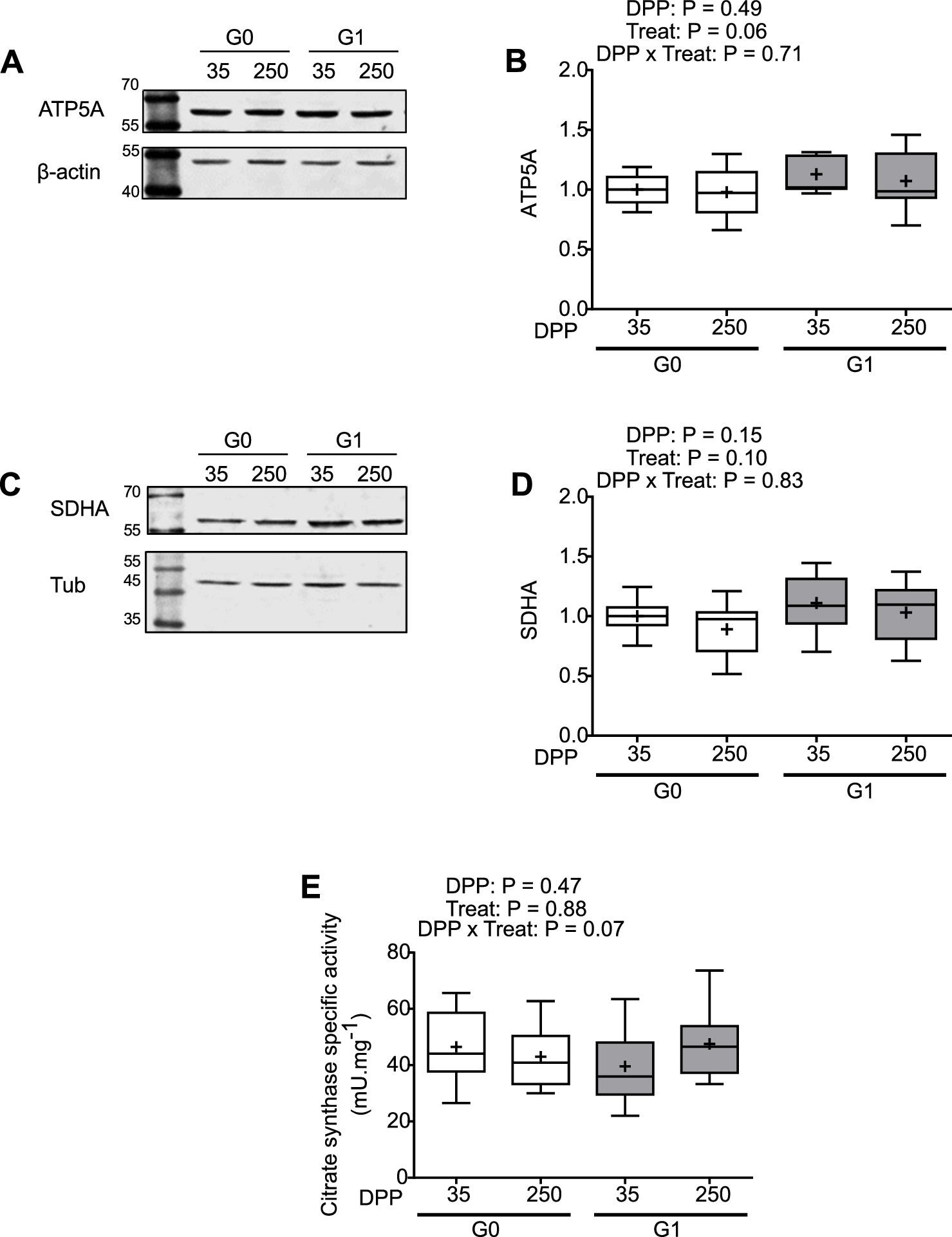

Supplement: S3 Fig — (A and C) Representative western blots of ATP synthase subunit α (ATP5A) and succinate dehydrogenase subunit A (SDHA) in liver homogenates of cows from both G0 and G1 groups at 35 and 250 DPP. β-actin and tubulin were used as loading controls. (B and D) Quantification by densitometry of ATP5A and SDHA levels normalized with the respective loading controls and expressed in relation to the average value of the G0 group at 35 DPP. (E) Citrate synthase specific activity was determined in liver homogenates of cows from the G0 and G1 group at 35 and 250 DPP. In box plots the box extends from the 25th to 75th percentile, the line in the middle of the box is the median, the cross is the mean and the whiskers represent the minimum and maximum values (N = 8–10). (TIF) [file pone.0213780.s003.tif]
