# Supplementary material for: Correction: Impaired hepatic mitochondrial function during early lactation in dairy cows: Association with protein lysine acetylation
Source: PLoS One. 2019 Apr 10;14(4):e0215395. doi: 10.1371/journal.pone.0215395 (PMC6457522; doi:10.1371/journal.pone.0215395)
Supplement: S2 File — (PDF) [file pone.0215395.s002.pdf]

PLOS ONE

RESEARCH ARTICLE

Impaired hepatic mitochondrial function during early lactation in dairy cows: Association with protein lysine acetylation

Mercedes García-Roche[1,2], Alberto Casal[3], Diego A. Mattiauda[3], Mateo Ceriani[3], Alejandra Jasinsky[3], Mauricio Mastrogiovanni[1], Andrés Trostchansky[1], Mariana Carriquiry[2]*, Adriana Cassina[1]*, Celia Quijano[1]*

1 Center for Free Radical and Biomedical Research (CEINBIO) and Departamento de Bioquímica, Facultad de Medicina, Universidad de la República, Montevideo, Uruguay, **2** Departamento de Producción Animal y Pasturas, Facultad de Agronomía, Universidad de la República, Montevideo, Uruguay, **3** Departamento de Producción Animal y Pasturas, Estación Experimental Mario A. Cassinoni, Facultad de Agronomía, Universidad de la República, Paysandú, Uruguay

* celia.quijano@gmail.com (CQ); acassina@fmed.edu.uy (AC); mariana.carriquiry@gmail.com (MC)

Check for updates

OPEN ACCESS

Citation: García-Roche M, Casal A, Mattiauda DA, Ceriani M, Jasinsky A, Mastrogiovanni M, et al. (2019) Impaired hepatic mitochondrial function during early lactation in dairy cows: Association with protein lysine acetylation. PLoS ONE 14(3): e0213780. https://doi.org/10.1371/journal.pone.0213780

Editor: Juan J Loor, University of Illinois, UNITED STATES

Received: August 7, 2018

Accepted: March 1, 2019

Published: March 14, 2019

Copyright: © 2019 García-Roche et al. This is an open access article distributed under the terms of the Creative Commons Attribution License, which permits unrestricted use, distribution, and reproduction in any medium, provided the original author and source are credited.

Data Availability Statement: All relevant data are within the manuscript and its Supporting Information files.

Funding: This work was supported by grants of Agencia Nacional de Investigación e Innovación (www.anii.org.uy):FSA_12612 awarded to M.C.; POS_NAC_2015_1_110049 awarded to M.G.R; POS_NAC_2014_1_102302 awarded to A. Casal. Espacio Interdisciplinario—Centros, UDELAR 2015 (www.ei.udelar.edu.uy): A. Cassina and C.Q; CSIC

Abstract

Early lactation is an energy-demanding period for dairy cows which may lead to negative energy balance, threatening animal health and consequently productivity. Herein we studied hepatic mitochondrial function in Holstein-Friesian multiparous dairy cows during lactation, under two different feeding strategies. During the first 180 days postpartum the cows were fed a total mixed ration (70% forage: 30% concentrate) *ad libitum* (non-grazing group, G0) or grazed *Festuca arundinacea* or *Mendicago sativa* plus supplementation (grazing group, G1). From 180 to 250 days postpartum, all cows grazed *Festuca arundinacea* and were supplemented with total mixed ration. Mitochondrial function was assessed measuring oxygen consumption rate in liver biopsies and revealed that maximum respiratory rate decreased significantly in grazing cows during early lactation, yet was unchanged in non-grazing cows during the lactation curve. While no differences could be found in mitochondrial content or oxidative stress markers, a significant increase in protein lysine acetylation was found in grazing cows during early lactation but not in cows from the non-grazing group. Mitochondrial acetylation positively correlated with liver triglycerides and β-hydroxybutyrate plasma levels, well-known markers of negative energy balance, while a negative correlation was found with the maximum respiratory rate and sirtuin 3 levels. To our knowledge this is the first report of mitochondrial function in liver biopsies of dairy cows during lactation. On the whole our results indicate that mitochondrial function is impaired during early lactation in grazing cows and that acetylation may account for changes in mitochondrial function in this period. Additionally, our results suggest that feeding total mixed ration during early lactation may be an efficient protective strategy.

grupos I+D 2014 (www.csic.edu.uy): 767, A. Cassina. The funders had no role in study design, data collection and analysis, decision to publish, or preparation of the manuscript.

Competing interests: The authors have declared that no competing interests exist.

Introduction

High yielding dairy cows are greatly challenged by the onset of lactation. Lactogenesis results in a dramatic increase in total energy requirements, and insufficient dry matter intake may lead to negative energy balance [1]. In addition to the physiological changes attributed to early lactation, the environment, particularly nutrition, is determinant in negative energy balance. Pasture-based systems are an economically advantageous alternative widely used in temperate regions [2]. However, pasture dry matter intake is highly dependent on cow physiology and behavior as well as sward characteristics and, in addition, may result in increased energy expenditure due to activity (grazing and walking)[2–4]. Previous studies have shown that limited pasture allowance may lead to higher mobilization of energy reserves, poor reproductive performance and limit the productive responses of dairy cows [5–8].

During early lactation, gut, liver, mammary gland and adipose tissue undergo adaptations to support lactation [9], in particular, a sharp increase in gluconeogenesis can be observed [10]. In order to meet energy demands and increase the availability of lactogenic precursors dairy cows mobilize body reserves [11,12], resulting in a decrease in body weight (BW) and body condition score (BCS). Excessive mobilization of adipose tissue triglycerides results in high levels of circulating non-esterified fatty acids (NEFA). The liver takes up NEFA [13]; that are either completely oxidized to carbon dioxide, partially oxidized to ketone bodies (an alternative fuel for non-hepatic tissues) or re-esterified into triglycerides and packaged into very low density lipoproteins for transport [14,15].

Imbalances in oxidation/re-esterification routes along with low synthesis and export rate of very low density lipoproteins can give place to hepatic steatosis also known as fatty liver [1,14,16], that can in turn progress towards steatohepatitis [17–19]. In humans and mice models with fatty liver decreased activity of the respiratory chain and β-oxidation enzymes, ultrastructure abnormalities, and increased mitochondrial reactive oxygen species (ROS) have been reported [18,20,21]. Although the molecular events behind mitochondrial impairment and ROS formation are not fully established, protein lysine acetylation appears as a relevant post-translational modification, capable of regulating both mitochondrial energy metabolism and redox status [22], and has been shown to increase in fatty liver of mice receiving a high-fat diet [20].

Although fatty liver syndrome is one of the most important metabolic diseases in high yielding dairy cows in early lactation [16,23], the pathogenesis of this disease is not thoroughly explored in ruminants; in particular the role of mitochondria has not been established. Pioneering studies assessing fatty acid oxidation in dairy cows showed that carnitine palmitoyl-transferase I activity and β-oxidation were impaired in cows with hepatic steatosis [24,25]. Recently, Gao *et al.* [26] reported a decrease in expression and activity in several subunits of respiratory complexes and of relevant regulators of mitochondrial biogenesis and fusion, in cows with signs of steatohepatitis. However much remains to be explored, in particular a systematic functional analysis of mitochondrial electron transport and oxidative phosphorylation during the different stages of the lactation curve is lacking.

Furthermore, given that most of hepatic ATP is synthesized by oxidative phosphorylation [27] the study of mitochondrial function is essential to understand the adaptations of energy metabolism during lactation. The high-energy demands of gluconeogenesis faced by the liver during this crucial period underscore the relevance of assessing mitochondrial function, yet few reports can be found on this matter.

In this work we aimed to quantify hepatic mitochondrial oxygen consumption rate in liver biopsies by high-resolution respirometry, of dairy cows during early and late lactation (35 and 250 days post partum, respectively); and to explore molecular mechanisms affecting

respiration, focusing on mitochondrial content, lipoperoxidation and protein acetylation. Two different feeding strategies (TMR and pasture-based diet) were used the first 180 days of lactation to assess if hepatic energy metabolism, in this crucial period, is affected by diet.

Materials and methods

Ethics statement

The use of animals and all animal procedures were approved by the Animal Experimentation Committee (CHEA) of the Universidad de la República, Montevideo, Uruguay (file number: 021130-001914-15).

Animals, feeding strategy and experimental design

Twenty-four multiparous Holstein-Friesian dairy cows calved in spring (664 ± 65 kg BW and 3.0 ± 0.4 units of BCS; 18/08/2015 ± 11 of calving date) grouped according to their due calving date, parity, BW and BCS were used in a randomized block design with two feeding strategies from calving to 180 ± 11 days postpartum (DPP): a non-grazing group (control group; G0) fed 100% of a total-mixed ration (TMR) *ad libitum* and a grazing group (G1) which grazed on pasture and received supplementation.

Cows in the non-grazing group (G0) (N = 12) were offered TMR once a day after the morning milking. The TMR had a forage to concentrate ratio of 70:30 (as fed basis) and was formulated according to NRC Dairy Model 2001 software [28] for a milk production target of 40 kg per day and 15–20% refusals. The TMR was composed by corn silage and moha (*Setaria italica*) hay or alfalfa haylage and a concentrate that included sorghum grain (22.6%), corn grain (6.8%), barley grain (4.4%) sunflower expeller (7.5%), soybean expeller (13.6%) and minerals and vitamins (1.7%). Cows were housed in a free stall facility (wood-frame barn) and wood shavings (> 10 cm) for bedding. Cows were allocated in three pens (8 x 22.6 m each; 4 cows per pen) and each pen had access to shade, water and a feeder (2.4 m high, 1.12 m wide in the top, 0.58 wide in the bottom and 0.50 m deep).

Cows in the grazing group (G1) (N = 12) grazed from 0 to 113 DPP a *Festuca arundinacea* pasture (2500 ± 490 kg DM per ha, 18 h of pasture access from 08:00 to 16:00 h and from 18:00 to 04:00 h) in a 7-d rotational system with a mean herbage allowance of 30 kg dry matter (DM) per cow per day (4 cm above ground level) and a chemical composition (DM basis) of 26.4% DM, 14.2% crude protein (CP), 54.7% neutral detergent fiber (NDF), 30.1% acid detergent fiber (ADF) and 1.58 Mcal per kg DM of net energy of lactation (NEL). In addition, after the morning milking, cows received, in individual feeders, 5.4 kg DM per cow per day of a concentrate containing corn grain (32%), barley grain (31%) and soybean expeller (32%) with a 87% DM, 16.8% CP, 28.5% NDF, 9.3% ADF and 1.83 Mcal per kg of NEL (DM basis). From 113 to 180 DPP they grazed a *Medicago sativa* pasture (1380 ± 328 kg DM per ha, 10 h of pasture access from 18:00 to 04:00 h) in a 7-d rotational system with a mean herbage allowance of 20 kg DM per cow per day (4 cm above ground level) and a chemical composition (DM basis) 26.4% DM, 23.3% CP, 30.1% NDF, 24.7% ADF and 1.68 Mcal per kg DM of NEL. During this period, after the morning milking, cows received, in the free stall facility, TMR (50% of offered TMR to G0 cows) composed by corn silage (23.3%), alfalfa haylage (19%), sorghum grain (20.8%), corn grain (11.8%), barley grain (11.5%), soybean expeller (11.8%) and minerals and vitamins (1.8%) with a chemical composition (DM basis) of 41.5% DM, 11.1% CP, 32.1% NDF, 22.0% ADF and 1.64 Mcal/kgDM of NEL. Diet change at 113 DPP was due to heat stress, since the temperature-humidity index exceeded the value of 72 for more than 5 consecutive hours and for 3 consecutive days [29].

After 180 DPP until the end of lactation, all cows (G0 and G1) grazed a *Festuca arundinacea* pasture (7-d rotational system; 11.5 h of pasture access from 16:30 to 04:00 h; with a herbage mass, above 4 cm of ground level, of 2340 ± 291 kg DM per ha and a herbage allowance of 20 kg DM per cow per day) with 28.3% DM, 10.1% CP, 56.6% NDF, 32.2% ADF and 1.48 Mcal per kg of NEL, DM basis) and were supplemented, after the morning milking, in the free stall facility, with TMR (50% of offered TMR to G0 cows at 180 DPP; 23.4% corn silage, 12.4% alfalfa hay, 28.3% sorghum grain, 11.5% corn grain, 11.1% barley grain, 11.5% soybean expeller and 1.8% minerals and vitamins) with 50% DM, 12.5% CP, 29.7% NDF, 18.8% ADF and 1.76 Mcal per kg of NEL (DM basis).

The proportion of pasture and TMR in the diet (DM basis) calculated for each treatment after the DM intake of TMR (based on difference between feed offered and refused) and pasture (based on NRC requirements) was determined. Diet was composed of 100% TMR from 0 to 180 DPP for G0 cows, and for G1 cows of 73.4% pasture and 26.6% concentrate from 0 to 113 DPP and 32.7% pasture and 67.3% TMR from 114 to 180 DPP; from 180 to 250 DPP diet was composed of 28% pasture and 72% TMR for all cows (G0 and G1). Nutrient composition of estimated diets is presented on Table 1.

Throughout the experiment, cows were milked twice a day and milk production was determined daily. Cow BCS (score 1 to 5)[30] and BW were recorded every two weeks.

Liver tissue collection and blood samples

Liver biopsies were collected using a 14-gauge biopsy needle (Tru-Core-II Automatic Biopsy Instrument; Angiotech) after the local intramuscular administration of 3 mL of 2% lidocaine HCl, as described previously [31] at -14, 35, 60, 100, 180 and 250 DPP for oxygen consumption rate measurements. Two dates representative of early and late lactation (35 and 250 DPP, respectively) were taken into consideration for further molecular studies. Biopsies for oxygen consumption rate measurements were cryopreserved as described previously [32]. Biopsies for

Table 1. Estimated nutrient composition of diets according to feeding strategy during lactation.

	G0[1]	G1		All cows
**Days postpartum**	**0 to 180**	**0 to 113**	**114 to 180**	**>180**
Chemical composition[2]				
Dry matter, %	43.1	42.4	36.5	43.9
Crude protein, %DM	12.9	14.9	15.1	11.8
Neutral detergent fiber, %DM	33.8	47.6	31.4	37.2
Acid detergent fiber, %DM	21.3	24.5	22.9	22.6
Net energy of lactation, Mcal/kg DM[3]	1.68	1.64	1.65	1.68
Metabolizable protein, g/d[3]	1854	1762	1914	1749

[1]Feeding strategies were a non-grazing group (control group; G0) fed 100% of a total-mixed ration (TMR) *ad libitum* and a grazing group (G1), which grazed on pasture and received supplementation.

[2]Diets were formulated to supply micronutrients according with requirements at all times of the lactation curve, thus they included a minerals and vitamin premix composed (% of DM or ppm, IU, g per animal) of 0.15% S, 19.31% Ca, 2.33% P, 2.98% Cl, 7.87% Na, 0.11% K, 3.59% Mg, 0.21 ppm Co, 5.7 ppm Cu, 12.9 ppm Fe, 8.8 ppm Mn, 0.08 ppm Se, 0.02 ppm Y, 18.7 Zn, 14.4 ppm chelated Zn, 5.04 ppm chelated Cu, 0.04 ppm chelated Se, 2,000.00 IU Vitamin A, 202.00 IU Vitamin D3, 2.10 IU Vitamin E, 12.16 ppm monensin, 0.56 g yeast, 0.28 g betaglucan, 0.28 g mannan-oligosaccharides.

[3]Net energy of lactation and metabolizable protein (MP) were estimated according to NRC (2001). Estimated MP balances indicates diets provided, at least 85% of MP requirement for both, G0 and G1, from 0 to 180 DPP and 95% of MP requirement after 180 DPP.

https://doi.org/10.1371/journal.pone.0213780.t001

western blot analysis and enzyme activity assays were immediately frozen in liquid nitrogen. All samples were stored at -80 ˚C until analysis. Although biopsies were taken from all cows, oxygen consumption measurements, mitochondrial isolation, Western Blots, triglycerides and activity measurements were performed for 8–10 cows of each treatment, due to tissue quantity.

Blood samples were collected at 35 and 250 DPP by venipuncture of the coccygeal vein using BD Vacutainer tubes with heparin (Becton Dickinson). Samples were centrifuged at 2000 g for 15 min at 4 ˚C within 1 hour after collection and plasma was stored at -20 ˚C until metabolite analyses were performed.

Mitochondrial isolation

Mitochondria were isolated as described previously [33]. Liver tissue was homogenized in homogenization buffer (250 mM sucrose, 50 mM Tris-HCl, 5 mM $MgCl_2$) with protease inhibitors (SigmaFast Protease Inhibitor Cocktail and 1 mM phenylmethylsulfonyl fluoride) and deacetylase inhibitors, (1 μM trichostatin A and 5 mM nicotinamide, pH 7.4) using a Potter-Elvehjem homogeneizer set to 600–1000 rpm. Homogenates were centrifuged at 800 g for 15 minutes twice to remove large pieces of tissue and nuclei. Then mitochondria were isolated by centrifugation at 11,000 g for 10 min. The pellet containing mitochondria was washed thoroughly three times centrifuging at 11,000 g and finally resuspended in 50–100 μL of 50 mM Tris HCl, 1 mM EDTA, 0.5% Triton-X-100 with protease and deacetylase inhibitors, pH 6.8. Subcellular fractions enriched in mitochondria were stored at -80 ˚C until analyzed. All procedures were carried out in the cold (4 ˚C). The enrichment and purity of the mitochondrial fraction was verified by Western blot (S1 Fig).

Mitochondrial oxygen consumption rate

Mitochondrial function was studied measuring oxygen consumption rate in a high-resolution respirometer OROBOROS Oxygraph—2k at 37 ˚C as described previously [32,34]. Electrodes were calibrated in modified MIR05 respiration medium (0.5 mM EGTA, 3mM $MgCl_2$•$6H_2O$, 60 mM MOPS, 20 mM taurine, 10 mM KH_2PO_4, 20 mM HEPES, 110 mM sucrose, 1 $g.L^{-1}$ BSA, pH 7.1) with a calculated saturated oxygen concentration of 191 μM at 100 kPa barometric pressure at 37 ˚C [34]. Respiratory rates (pmol $O_2.min^{-1}.mL^{-1}$) were calculated using the DatLab 4 analysis software. Liver biopsies (2–10 mg) were weighed, added to the chamber and oxygen consumption measurements were obtained before and after the sequential addition of specific substrates of the respiratory chain, 10 mM glutamate plus 5 mM malate (complex I) or 20 mM succinate (complex II), followed by 4 mM adenosine diphosphate (ADP), 2 μM oligomycin (ATP synthase inhibitor), 2–4 μM carbonyl cyanide-p-trifluoromethoxyphenylhydrazone (FCCP, an uncoupler of oxidative phosphorylation). Maximum uncoupling was obtained titrating FCCP concentrations used in the assay. Finally, respiration was inhibited with 0.5 μM rotenone (complex I inhibitor) or 2.5 μM antimycin A (complex III inhibitor).

All respiratory parameters and indices were obtained as described in [27,32]. Briefly, the non-mitochondrial oxygen consumption rate was determined after adding antimycin A or rotenone and subtracted from all other values before calculating the respiratory parameters. State 4 respiration was the baseline measurement obtained with substrates before the addition of ADP and state 3 respiration was determined after addition of ADP. Oligomycin-resistant respiration (ATP-independent) was measured after oligomycin injection and oligomycin-sensitive respiration (ATP-dependent) was calculated as the difference between state 3 and oligomycin-resistant respiration. Maximum respiratory capacity was determined after the addition of FCCP.

Citrate synthase activity

Citrate synthase is a constitutive mitochondrial enzyme frequently used as a marker for mitochondrial content [35]. To determine its activity, liver tissue (100 mg) was homogenized using a Potter-Elvehjem homogenizer in 10 volumes of homogenization buffer (5 mM KH_2PO_4, 1 mM EGTA, 5 mM MOPS, 300 mM sucrose at pH 7.1). Enzyme activity was measured in homogenates following the formation of 5-thio-2-nitrobenzoic acid at λ = 412 nm (ε_{412} = 13,700 $M^{-1}.cm^{-1}$) in the presence of 20 mM Tris-HCl pH 8, 300 μM acetyl-CoA, 500 μM oxaloacetate, 100 μM 5,5'-dithio-bis (2-nitrobenzoic acid), and 60 $\mu g.mL^{-1}$ of liver protein [35]. Specific activity was calculated after determining the protein concentration of the samples with the Bradford assay using bovine serum albumin as standard [36].

Western blots

Liver tissue (10–20 mg) was disrupted using a Potter-Elvehjem homogenizer in 10 volumes of cold lysis buffer (150 mM NaCl, 2 mM EDTA, 2 mM EGTA, 1% Triton X-100, 0.1% SDS with protease and deacetylase inhibitors).

After homogenization, samples were placed on a rotator at 4 ˚C for 1 hour. Samples for western blots with antibodies against mitochondrial respiratory chain subunits were spun at 12,000 g for 10 min at 4 ˚C and the supernatants containing soluble proteins were stored. The last step was avoided when preparing samples for western blots with antibodies against acetyl lysine (AcK) and 4-hydroxynonenal (4-HNE). Protein content was determined with the Bradford assay using bovine serum albumin as standard [36] and samples were kept at -80 ˚C until analyzed.

Liver homogenates (30–40 μg) and subcellular fractions enriched in mitochondria (20 μg) were resolved in 10 to 12% Tris-Glycine-SDS polyacrylamide gels (SDS/PAGE), along with protein ladders (LI-COR Biosciences 928–60000 or Thermo Fisher Scientific 26616), and proteins were transferred overnight to nitrocellulose membranes. Membranes were blocked with blocking buffer (Tris buffered saline with 0.1% Tween 20 and 0.5% skimmed milk) and incubated overnight at 4 ˚C with primary antibodies against: GAPDH (1:1000, Abcam, ab9484), β-actin (1:1000, Santa Cruz, sc-81178), α-tubulin (1:1000, Santa Cruz, sc-8035), succinate dehydrogenase subunit A (SDHA, 1:2000, Abcam, ab14715), α subunit of ATP synthase (ATP5A, 1:1000, Abcam, ab14748), acetylated lysine (1:1000, Cell Signaling Technology, 9441), protein-4-HNE adducts (1:1000, Abcam, ab46544), sirtuin 3 (1:1000, Cell Signaling Technology, 5490), sirtuin 5 (1:1000, Cell Signaling Technology, 8782), 3-nitrotyrosine (1:1000, a kind gift from Dr. Rafael Radi, CEINBIO, Departamento de Bioquímica, Facultad de Medicina, Universidad de la República, Uruguay) and histone H3 (1:1000, Cell Signaling Technology, 4620). Membranes were washed and probed with secondary antibodies from LI-COR Biosciences: anti-mouse (1:10,000, IRDye 680, 926–68070), anti-rabbit (1:20,000, IRDye 800, 926–32211) or anti-goat (1:20,000, IRDye 800, 925–32214). Immunoreactive proteins were detected with an infrared fluorescence detection system (Odyssey, LI-COR Biosciences) and bands were quantified by densitometry with ImageStudio software (LI-COR Biosciences).

Hepatic triglycerides

Lipids were extracted from liver homogenates (6 $mg.mL^{-1}$), in hexane/isopropanol/1 M acetic acid (30:20:2, v/v/v), in a 1:2.5 sample to solvent ratio. After vortexing for 30 seconds, 2.5 volumes of hexane were added, vortexed and the mixture was centrifuged at 1800 g for 5 min at 4 ˚C and the organic phase, containing the lipids, was separated from the aqueous phase. Hexane was added to the aqueous phase and centrifuged at 1800 g for 5 min at 4 ˚C to increase lipid recovery. Before extraction 1-dodecanol (Sigma, 75544) was added to the samples and used as

an internal standard for normalization in semi-quantification analyses. Finally hexane phases were pooled and subjected to solvent evaporation under vacuum in a RapidVap Vacuum Evaporation System (Labconco) [37].

Lipid extracts were dissolved in chloroform and spotted manually on thin layer chromatography plates using a microsyringe (Hamilton) along with the internal standard and a triglyceride standard. The triglyceride standard was an olive oil sample containing more than 98% triglycerides, characterized at the Instituto Nacional de Investigación Agropecuaria (INIA), Uruguay, by gas chromatography under the reference of the Intenational Oil Council (Norma COI/T.20/Doc. n˚ 24 2001). Lipids were separated using hexane/diethyl-ether/acetic acid (80:20:1, v/v/v) as mobile phase [38] and lipid bands visualized after spraying with 5% sulphuric acid (v/v) in ethanol and heating. Densitometry quantification analysis of the bands was performed using ImageJ software.

Plasma biochemical assays

Plasma β-hydroxybutyrate concentrations were determined with a kit from Randox Laboratories Ltd. following manufacturer instructions. The assay measures NADH formation spectrophotometrically at 340 nm during β-hydroxybutyrate dehydrogenase catalyzed oxidation of β-hydroxybutyrate to acetoacetate [39].

Plasma NEFA concentrations were determined spectrophotometrically with a kit from FUJIFILM Wako Diagnostics, following manufacturer instructions. In this method, NEFA incubated with acyl-CoA synthetase and ATP yield acyl-CoA. Acyl-CoA is oxidized in a reaction catalyzed by acyl-CoA oxidase producing hydrogen peroxide, which in the presence of peroxidase forms a purple colored end-product with an absorption maximum at 550 nm [40].

Plasma aspartate aminotransferase (AST) catalytic activity was determined with a kit from Biosystems, following manufacturer instructions. Aspartate aminotransferase catalyzes the transference of an amino group from aspartate to 2-oxoglutarate, forming oxaloacetate and glutamate. The assay measures the decrease of NADH spectrophotometrically at 340 nm in the malate dehydrogenase coupled reaction [41].

All plasma biochemical assays were performed using a Vitalab Selectra 2 autoanalyzer (Vital Scientific).

Statistical analyses

Data were analyzed in a randomized block design using the SAS System program (SAS Academic Edition; SAS Institute Inc., Cary, NC, USA). Univariate and linear regression analyses were performed with all variables to identify outliers and inconsistencies and to verify normality of residuals. Outliers were removed when the residual had a Studentized residual < -4 or > 4. In the case of β-hydroxybutyrate and AST natural logarithmic transformations were performed and back transformed values were used to calculate means, standard errors and graph data. Data were analyzed as repeated measures using the MIXED procedure, the model included treatment, DPP and their interaction as fixed effects, block and cow as random effects and calving date, initial BW and BCS as covariates when $P < 0.20$. Tukey-Kramer tests were conducted to analyze differences between groups. BCS was analyzed using the GENMOD procedure, the model included treatment, DPP and their interaction as fixed effects. Means were considered to differ when $P < 0.05$ and indicated with different letters in tables or asterisks in graphs ($^{*}P < 0.05$, $^{**}P < 0.01$, $^{***}P < 0.001$ and $^{****}P < 0.0001$) and trends were identified when $0.05 < P < 0.10$. Correlation analyses between variables were performed using the CORR procedure.

Results

Productive and metabolic parameters

Since our aim was to compare hepatic mitochondrial function during early and late lactation we verified the productive parameters of the animals at this two dates of the lactation curve. No interaction between DPP and feeding strategy was found for milk yield or BCS (Table 2). As expected milk production was higher ($P < 0.001$) while BCS was lower ($P < 0.001$) at 35 than at 250 DPP, but no differences were observed between feeding strategies and (Table 2).

To assess the general metabolic status of the cows, under different diets, during lactation we measured liver triglycerides, concentrations of β-hydroxybutyrate and NEFA in plasma (Fig 1) and AST activity in plasma.

The interaction of DPP and treatment was significant for plasma β-hydroxybutyrate and for liver triglyceride (DPP X Treat: $P< 0.05$), as average concentrations were two-fold higher during early lactation for G1 versus G0 cows (Fig 1A and 1B), while remaining unchanged by diet in late lactation. These observations suggest that feeding strategy impacts fatty acid metabolism in lactation. No significant interactions were found between DPP and treatment for plasma NEFA (Fig 1C), but values were higher during early than late lactation ($P < 0.05$). Additionally, the correlation coefficient between β-hydroxybutyrate and NEFA was positive and significant and tended to be significant between triglyceride and β-hydroxybutyrate (Fig 1D and 1E), while the correlation between liver triglyceride and NEFA was not significant.

We then measured AST activity in plasma to assess liver damage. No interaction between treatment and dates was found, neither significant differences between groups or DPP (50 ± 6 $U.L^{-1}$ in the G0 group versus 63 ± 6 $U.L^{-1}$ in the G1 group at 35 DPP; and 68 ± 6 $U.L^{-1}$ in the G0 group versus 60 ± 6 in the G1 group at 250 DPP; N = 12).

Overall our results indicate that average values of β-hydroxybutyrate and NEFA of the cows in our study were below pathological threshold (<1.2 $mmol.L^{-1}$ and <1 $mmol.L^{-1}$ respectively) [42]. Nevertheless, these markers of negative energy balance were higher during early than late lactation [42], in particular in the G1 group. Liver triglycerides indicated that in average cows had moderate fatty liver (triglyceride 5–10% of wet weight [16,42,43]) during early lactation, and mild fatty liver in late lactation (triglyceride 1–5% of wet weight [16,42,43]). Average values of AST activity were below the cut off value (<110 $U.L^{-1}$) for both groups during both lactation moments [43].

Table 2. Productive parameters.

	Treat	DPP		P-value		
		35	250	DPP	Treat	DPP x Treat
Milk yield (kg/d)	G0	35.8 ± 0.8[a]	19.2 ± 0.8[b]	< 0.001	0.34	0.59
	G1	36.2 ± 0.8[a]	20.3 ± 0.8[b]			
BCS (units)	G0	2.44 ± 0.05[b]	2.71 ± 0.05[a]	< 0.001	1.00	0.77
	G1	2.46 ± 0.05[b]	2.69 ± 0.05[a]			

Average weekly milk yield and body condition score (BCS) were determined at 35 and 250 days postpartum (DPP). Milk yield and body condition score (BCS) were determined at 35 and 250 days postpartum (DPP) in cows under two different feeding strategies or treatments (Treat), G1 and G0. All data is shown as least square means ± standard error (N = 12).

[ab] Different letters denote differences between rows and columns ($P < 0.05$) according to Tukey-Kramer test. G0: Cows were fed TMR *ad libitum* from calving to 180 DPP G1: Cows grazed *Festuca arundinacea* plus a commercial concentrate or *Medicago* supplemented with TMR (50% of G0 offer), depending on heat stress conditions, from calving to 180 DPP. From 180 to 250 DPP both groups grazed *Medicago sativa* and were supplemented with TMR (50% of G0 offer at 180 DPP).

https://doi.org/10.1371/journal.pone.0213780.t002

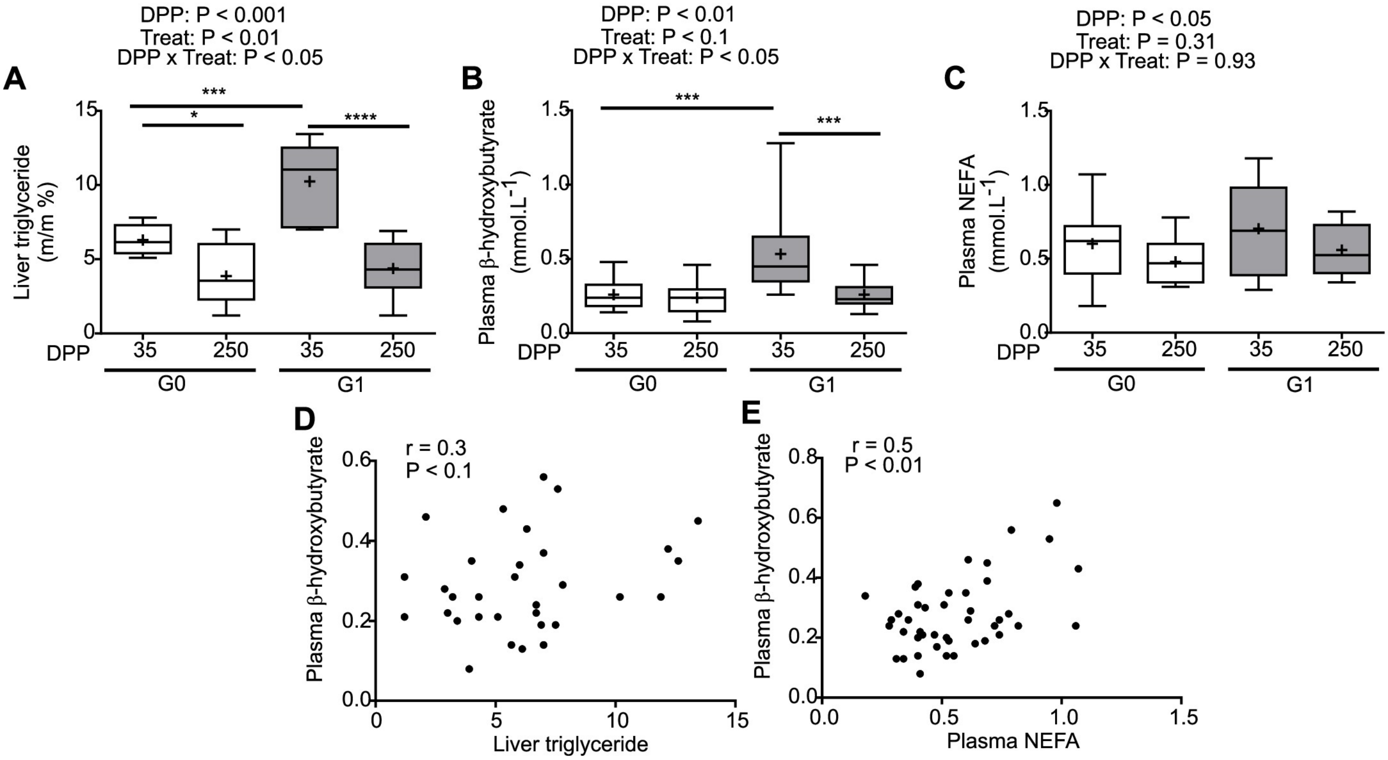

Fig 1. Metabolic parameters during lactation. Liver biopsies and blood samples were obtained at 35 and 250 DPP from cows in the G0 (white) and G1 (grey) groups. Graphs show the concentrations of **(A)** liver triglyceride, **(B)** plasma β-hydroxybutyrate and **(C)** plasma NEFA. Results are shown with box plots, the box extends from the 25th to 75th percentile, the line in the middle of the box is the median, the cross is the mean and the whiskers represent the minimum and maximum values (N = 8–12), * $P < 0.05$, *** $P < 0.001$, **** $P < 0.0001$. In graphs (**D**) and (**E**) the correlations between β-hydroxybutyrate, triglycerides and NEFA are shown (N = 8–12). G0: Cows were fed TMR *ad libitum* from calving to 180 DPP. G1: Cows grazed *Festuca arundinacea* plus a commercial concentrate or *Medicago* supplemented with TMR (50% of G0 offer), depending on heat stress conditions, from calving to 180 DPP. From 180 to 250 DPP both groups grazed *Medicago sativa* and were supplemented with TMR (50% of G0 offer at 180 DPP).

https://doi.org/10.1371/journal.pone.0213780.g001

Mitochondrial function

Since fatty acid catabolism occurs in mitochondria in strict coordination with energy demands and relies heavily on mitochondrial function [44], respiratory analyses were carried out in liver biopsies, to assess electron transport chain activity and oxidative phosphorylation. Oxygen consumption rates were measured after addition of substrates of the respiratory chain, ADP, inhibitors and an uncoupler of oxidative phosphorylation (Fig 2 and Tables 3 and 4).

While complex I dependent respiration remained unchanged in G0 cows between the different DPP (Fig 2A), it was considerably lower in early lactation than in late lactation for the G1 group (Fig 2B). Significant interactions ($P < 0.05$) between DPP and treatment were found for complex I respiratory parameters related to respiratory chain activity and ATP synthesis. State 3, maximum and oligomycin sensitive respiration decreased in hepatic biopsies from G1 cows during early lactation when compared with late lactation, while remaining unchanged for G0 at the different dates (Table 3 and Fig 2C). Assessment of the maximum respiratory rate at different moments during the lactation curve (Fig 2D) revealed that this parameter had similar values from -14 to 180 DPP, becoming significantly higher at 250 DPP; and that significant differences between treatments could be detected only at 35 DPP. Maximum respiratory rate correlated negatively with liver triglyceride (Fig 2G) and β-hydroxybutyrate (Fig 2H), suggesting that decreased mitochondrial function is linked to liver steatosis and ketone body synthesis in dairy cows.

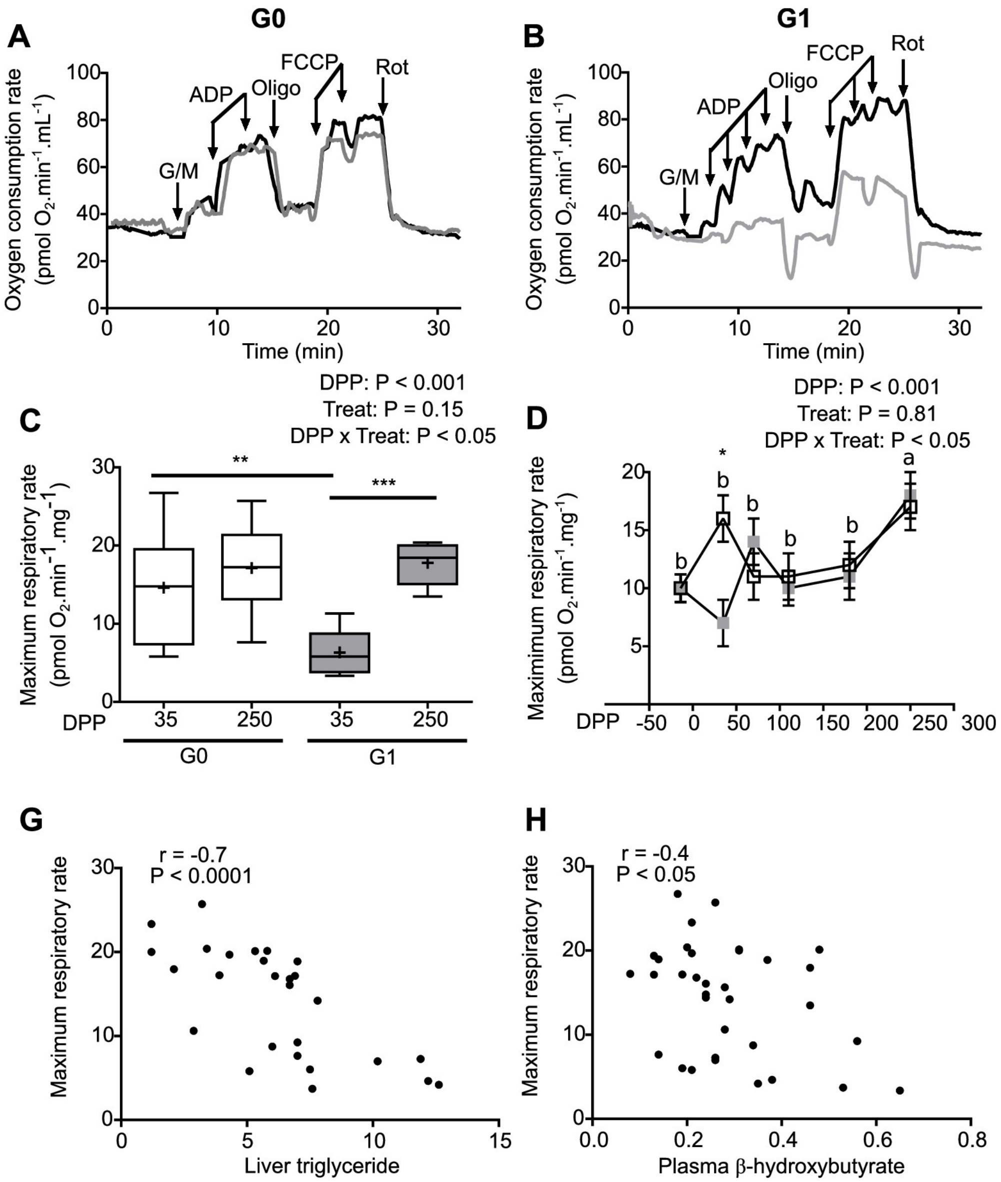

Fig 2. Mitochondrial function decreases in pasture-fed dairy cows during early lactation. Oxygen consumption rates were measured in liver biopsies before and after the sequential addition of 10 mM glutamate and 5 mM malate (Glu/Mal), 4 μM ADP, 2 μM oligomycin (Oligo), up to 4 μM FCCP and 0.5 μM rotenone (Rot). **(A and B)** Show representative traces of oxygen consumption rates obtained for liver biopsies of cows in the G0 group **(A)** and G1 group **(B)** at 35 DPP (grey) and 250 DPP (black). **(C)** Maximum respiratory rate, obtained from oxygen consumption rate measurements performed as described in A and B, of liver biopsies from cows in the G0 (white) and G1 (grey) groups. The box extends from the 25th to 75th percentile, the line in the middle of the box is the median, the cross is the mean and the whiskers represent the minimum and maximum values (N = 9–10), ** $P < 0.01$, and *** $P < 0.001$. **(D)** Maximum respiratory rate of liver biopsies obtained at different points during the lactation curve for both G0 (empty squares) and G1 (grey squares) cows. Data represent least square means ± SEM (N = 9–10). Different letters denote differences between dates ($P < 0.05$) and * denotes a difference between treatments ($P < 0.05$) according to Tukey-Kramer test. **(G)** and **(H)** show the correlation between maximum respiratory rate and liver triglyceride and plasma β-hydroxybutyrate, respectively (N = 8–12). G0: Cows were fed TMR *ad libitum* from calving to 180 DPP. G1: Cows grazed *Festuca arundinacea* plus a commercial concentrate or *Medicago* supplemented with TMR (50% of G0 offer), depending on heat stress conditions, from calving to 180 DPP. From 180 to 250 DPP both groups grazed *Medicago sativa* and were supplemented with TMR (50% of G0 offer at 180 DPP).

https://doi.org/10.1371/journal.pone.0213780.g002

Table 3. Complex I-dependent respiratory parameters.

Respiratory parameters	Treat	DPP		P-value		
		35	250	DPP	Treat	DPP x Treat
State 3	G0	12 ± 2^{ab}	15 ± 2^{a}	< 0.001	0.34	< 0.05
	G1	7 ± 2^{b}	16 ± 2^{a}			
State 4	G0	4 ± 1^{c}	6 ± 1^{b}	< 0.001	0.21	< 0.05
	G1	3 ± 1^{c}	9 ± 1^{a}			
Maximum	G0	15 ± 2^{a}	17 ± 2^{a}	< 0.001	0.15	<0.05
	G1	8 ± 2^{b}	18 ± 2^{a}			
Oligomycin-resistant	G0	3 ± 1^{c}	8 ± 1^{b}	< 0.001	0.22	0.06
	G1	3 ± 1^{c}	11 ± 1^{a}			
Oligomycin-sensitive	G0	7 ± 1^{a}	7 ± 1^{a}	0.09	0.17	<0.05
	G1	3 ± 1^{b}	7 ± 1^{a}			
Non-mitochondrial	G0	8 ± 0.8^{a}	6 ± 0.8^{b}	< 0.01	0.66	0.95
	G1	8 ± 0.8^{a}	6 ± 0.8^{b}			

Respiratory parameters were determined at 35 and 250 DPP in biopsies from cows under two different feeding strategies or treatments (Treat), G1 and G0. Oxygen consumption rates were measured after the sequential addition of 10 mM glutamate and 5 mM malate, 4 μM ADP, 2 μM oligomycin, up to 4 μM FCCP and 0.5 μM rotenone (as shown in Fig 2). Respiratory parameters were calculated as described in Materials and Methods. All data is shown as least square means ± standard error (N = 8–10). Oxygen consumption rates are expressed as pmol O_2.min^{-1}.mg wet weight^{-1}.

[abc] Different letters denote differences between rows and columns (P < 0.05) according to Tukey-Kramer test. G0: Cows were fed TMR *ad libitum* from calving to 180 DPP. G1: Cows grazed *Festuca arundinacea* plus a commercial concentrate or *Medicago* supplemented with TMR (50% of G0 offer), depending on heat stress conditions, from calving to 180 DPP. From 180 to 250 DPP both groups grazed *Medicago sativa* and were supplemented with TMR (50% of G0 offer at 180 DPP).

https://doi.org/10.1371/journal.pone.0213780.t003

Interestingly, the interaction between dates and treatments for state 4 was significant (P < 0.05) and presented a trend for oligomycin-resistant respiration (P = 0.06) (Table 3). These parameters are associated with events that dissipate the mitochondrial membrane potential (e.g. ion transport across the inner mitochondrial membrane) but not with ATP synthesis. Non-mitochondrial oxygen consumption was increased in early lactation with respect to late lactation (P < 0.01) (Table 3); while no differences were detected between treatments (Table 3).

No interaction between DPP and treatment was found for respiratory parameters obtained with complex II substrates were used (Table 4). However, as observed for complex I, maximum respiratory rate was lower (P <0.05) while non-mitochondrial oxygen consumption was higher (P< 0.001) at 35 DPP than 250 DPP (Table 4).

Oxidative stress markers

Since non-mitochondrial oxygen consumption has been associated with reactive oxygen species (ROS) formation [45,46], we looked for oxidative modifications in the tissue. We analyzed the levels of 4-HNE-protein adducts, a product of lipid peroxidation [47], both in homogenates and subcellular fractions enriched in mitochondria and no significant interaction between DPP and treatment were found; nor were differences between dates or treatments detected (Fig 3). Levels of 4-HNE-protein adduct did not correlate with maximum respiratory capacity, however, the correlation between 4HNE-protein adducts and non-mitochondrial oxygen consumption rate was positive and significant (r = 0.4, P < 0.05).

We also tried to measure 3-nitrotyrosine levels, a marker of oxidative events involving nitric oxide derived radicals and oxidant species [48]; but protein tyrosine nitration could not be

Table 4. Complex II-dependent respiratory parameters.

Respiratory parameters	Treat	DPP		P-value		
		35	250	DPP	Treat	DPP x Treat
State 3	G0	43 ± 6	56 ± 6	0.08	0.76	0.79
	G1	43 ± 6	52 ± 6			
State 4	G0	28 ± 4	37 ± 4	0.08	0.97	0.83
	G1	30 ± 4	36 ± 4			
Maximum	G0	57 ± 9[b]	77 ± 8[a]	< 0.05	0.95	0.96
	G1	58 ± 9[b]	77 ± 8[a]			
Oligomycin-resistant	G0	34 ± 5	42 ± 4	0.09	0.85	0.74
	G1	33 ± 5	42 ± 4			
Oligomycin-sensitive	G0	10 ± 2	14 ± 2	0.09	0.23	0.87
	G1	7 ± 2	10 ± 2			
Non-mitochondrial	G0	8 ± 1[a]	4 ± 1[b]	< 0.001	0.43	0.78
	G1	8 ± 1[a]	4 ± 1[b]			

Respiratory parameters were determined at 35 and 250 DPP in biopsies from cows under two different feeding strategies or treatments (Treat), G1 and G0. Oxygen consumption rate measurements of liver biopsies were obtained after addition of 20 mM succinate, 4 μM ADP, 2 μM oligomycin, up to 4 μM FCCP and 2.5 μM antimycin. Respiratory parameters were calculated as described in Materials and Methods. All data is shown as least square means ± standard error (N = 8–10). Oxygen consumption rates are expressed as pmol O_2.min^{-1}.mg wet weight^{-1}.

[ab]Different letters denote differences between rows and columns (P < 0.05) according to Tukey-Kramer test. G0: Cows were fed TMR *ad libitum* from calving to 180 DPP. G1: Cows grazed *Festuca arundinacea* plus a commercial concentrate or *Medicago* supplemented with TMR (50% of G0 offer), depending on heat stress conditions, from calving to 180 DPP. From 180 to 250 DPP both groups grazed *Medicago sativa* and were supplemented with TMR (50% of G0 offer at 180 DPP).

https://doi.org/10.1371/journal.pone.0213780.t004

identified in the tissue (S2 Fig). Controls were performed exposing liver homogenates to the strong oxidizing and nitrating agent peroxynitrite (S2 Fig).

Mitochondrial content

Since changes in mitochondrial content could be accountable for changes in mitochondrial respiration rates, levels of mitochondrial proteins were assessed (i.e. ATP5A, SDHA) and citrate synthase activity was measured in whole tissue homogenates. No interaction was found between DPP and treatments and there were no significant differences in the levels of these mitochondrial proteins or in citrate synthase activity, between dates or between treatments either (S3 Fig). Correlations between these three markers and maximum respiratory rate were not significant.

Protein lysine acetylation

Since neither an increase in oxidative stress nor changes in mitochondrial content could explain the decay in mitochondrial respiration we looked into protein lysine acetylation, since it has been recently described as a key regulator of energy metabolism [49–51]. Evaluation of AcK levels in isolated mitochondria (Fig 4) showed the existence of a significant interaction of DPP and treatment (P < 0.01). Protein acetylation was higher during early than late lactation in G1 (70% increase approximately), while remaining unchanged in G0 cows during lactation (Fig 4B). Mitochondrial AcK levels displayed a significant and positive correlation with liver triglycerides (Fig 4C) and with β-hydroxybutyrate (Fig 4D), in agreement with previous reports on regulation of liver lipid metabolism by acetylation [49]. In addition mitochondrial AcK levels and maximum respiratory rate presented a negative correlation (Fig 4E) suggesting

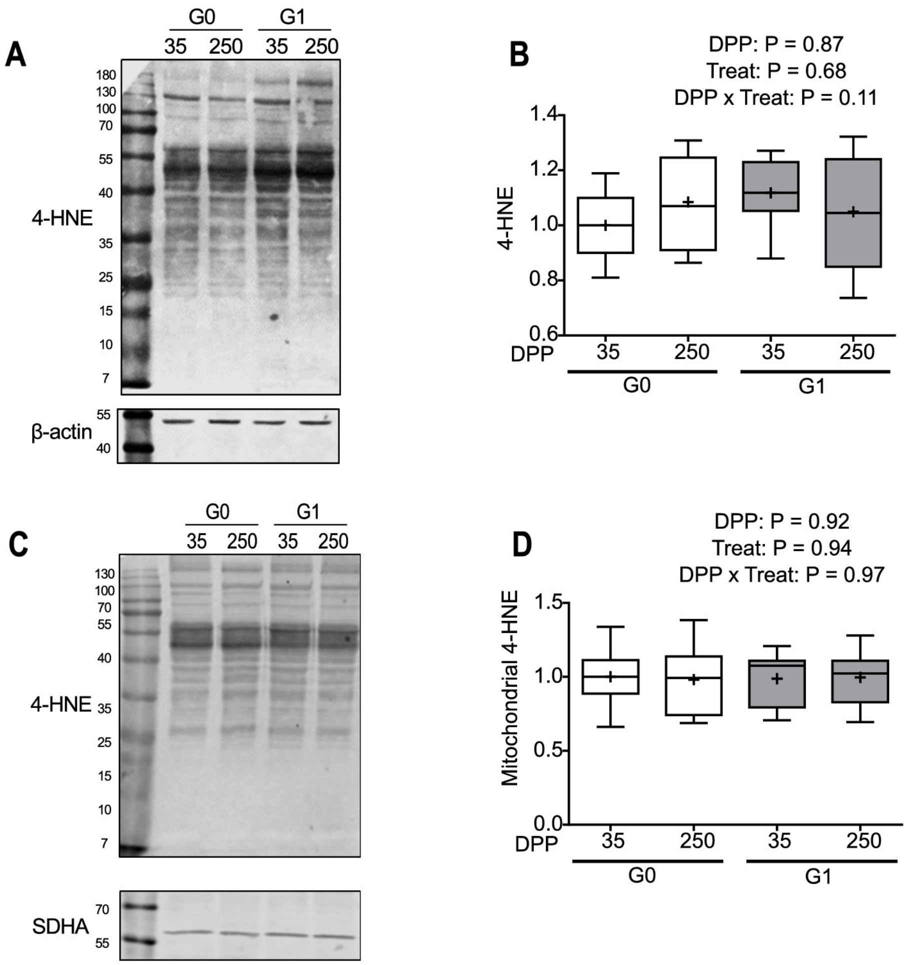

Fig 3. Evaluation of 4-HNE-protein adducts formation in liver homogenates and mitochondria. (A and C) Representative western blots of 4-HNE-protein adducts in liver homogenates **(A)** and isolated mitochondria **(C)** from cows in the G0 and G1 groups at 35 and 250 DPP; β-actin and SDHA were used as loading controls, respectively. **(B and D)** Quantification by densitometry of 4-HNE-protein adduct levels normalized by protein levels of loading control and expressed in relation to the average value of the G0 group at 35 DPP. In box plots the box extends from the 25th to 75th percentile, the line in the middle of the box is the median, the cross is the mean and the whiskers represent the minimum and maximum values (N = 8–10). G0: Cows were fed TMR *ad libitum* from calving to 180 DPP. G1: Cows grazed *Festuca arundinacea* plus a commercial concentrate or *Medicago* supplemented with TMR (50% of G0 offer), depending on heat stress conditions, from calving to 180 DPP. From 180 to 250 DPP both groups grazed *Medicago sativa* and were supplemented with TMR (50% of G0 offer at 180 DPP).

https://doi.org/10.1371/journal.pone.0213780.g003

that AcK could be responsible for the decrease in mitochondrial function observed during early lactation in pasture fed cows.

To further assess the extent of protein acetylation in the liver we studied AcK levels in tissue homogenates (Fig 5). The interaction between DPP and treatment tended to be significant ($P < 0.1$). AcK levels in liver homogenates of G0 cows were 30% lower during late lactation compared to early lactation and to G1 cows in the same period. No significant correlation was found between acetylated lysine levels in liver homogenates and maximum respiratory rate. These results indicate that the differences in mitochondrial acetylation are organelle specific and not a consequence of general changes in acetylation in the tissue.

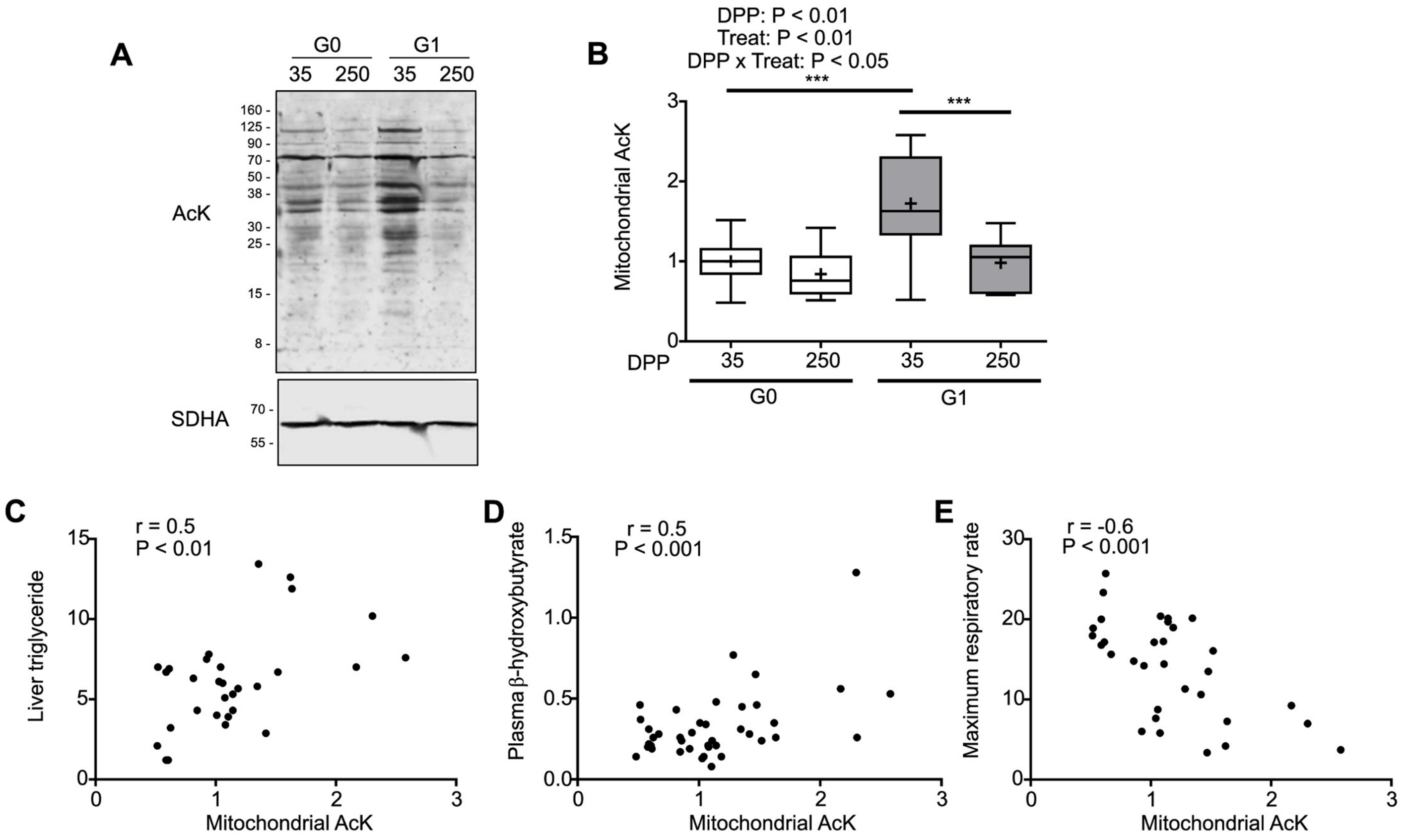

Fig 4. Protein acetylation increases in liver mitochondria from pasture-fed dairy cows during early lactation. **(A)** Representative western blots for AcK and SDHA (loading control) in liver subcellular fractions enriched in mitochondria from cows of both G0 and G1 groups at 35 and 250 DPP. **(B)** Independent western blots were quantified by densitometry. AcK levels were normalized with the loading control and expressed in relation to the average value of the G0 group at 35 DPP. The box extends from the 25th to 75th percentile, the line in the middle of the box is the median, the cross is the mean and the whiskers represent the minimum and maximum values (N = 10), *** $P < 0.001$. **(C)**, **(D)** and **(E)** show the correlations between AcK levels and liver triglyceride, plasma β-hydroxybutyrate and maximum respiratory rate, respectively (N = 8–10). G0: Cows were fed TMR *ad libitum* from calving to 180 DPP. G1: Cows grazed *Festuca arundinacea* plus a commercial concentrate or *Medicago* supplemented with TMR (50% of G0 offer), depending on heat stress conditions, from calving to 180 DPP. From 180 to 250 DPP both groups grazed *Medicago sativa* and were supplemented with TMR (50% of G0 offer at 180 DPP).

https://doi.org/10.1371/journal.pone.0213780.g004

Mitochondrial sirtuins

We then studied the levels of the mitochondrial sirtuins 3 and 5 (Fig 6). These enzymes catalyze the NAD dependent deacetylation of mitochondrial proteins and have been reported to regulate the activity of proteins involved in oxidative phosphorylation [52,53].

No significant interactions of treatment and dates were observed for either of the sirtuins (Fig 6B and 6D). Besides, in the case of sirtuin 5 no differences were observed between DPP or treatments (Fig 6A and 6B). However, sirtuin 3 levels were lower for the G1 cows than the G0 cows in both lactation moments ($P < 0.05$) (Fig 6C and 6D). The correlation between sirtuin 3 levels and mitochondrial AcK was negative and significant (Fig 6E) while a positive and significant correlation was found between sirtuin 3 levels and mitochondrial maximum respiratory rate (Fig 6F). These correlations suggest that a decrease in sirtuin 3 levels could be behind the observed increase in protein acetylation and the decay in mitochondrial function.

Discussion

This study presents evidence of impairment in hepatic mitochondrial respiration during early lactation in pasture-fed cows, with increased markers of negative energy balance. Several

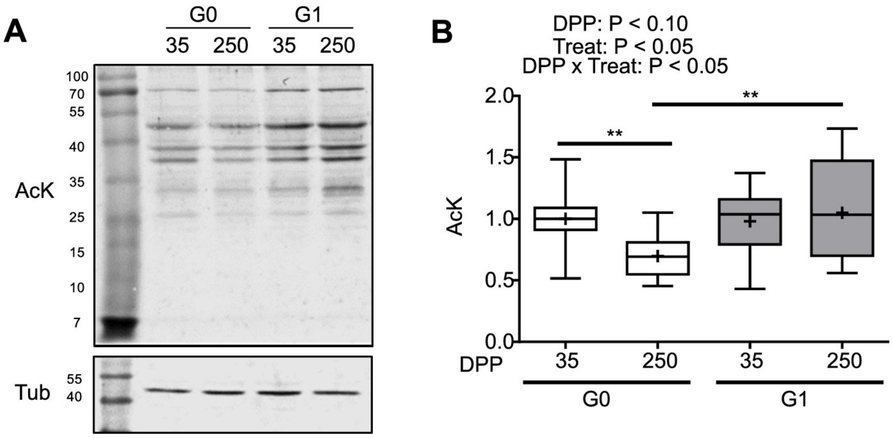

Fig 5. Protein lysine acetylation in liver homogenates. **(A)** Representative Western blot of AcK levels in liver homogenates from cows of both G0 and G1 groups at 35 and 250 DPP; tubulin was used as loading control. **(B)** Quantification by densitometry of total AcK levels normalized with the loading control and expressed in relation to the average value of the G0 group at 35 DPP. The box extends from the 25th to 75th percentile, the line in the middle of the box is the median, the cross is the mean and the whiskers represent the minimum and maximum values (N = 10). ** $P < 0.01$. G0: Cows were fed TMR *ad libitum* from calving to 180 DPP. G1: Cows grazed *Festuca arundinacea* plus a commercial concentrate or *Medicago* supplemented with TMR (50% of G0 offer), depending on heat stress conditions, from calving to 180 DPP. From 180 to 250 DPP both groups grazed *Medicago sativa* and were supplemented with TMR (50% of G0 offer at 180 DPP).

https://doi.org/10.1371/journal.pone.0213780.g005

respiratory parameters indicative of mitochondrial function (state 3, maximum, oligomycine sensitive respiration) were decreased in early lactation in pasture fed cows, but not in cows in the TMR diet. In particular, maximum respiratory rates were affected, indicating a decrease in the capacity to adapt to energy demands or to withstand damaging insults [27,32]. Additionally we observed that maximum respiratory rate correlated negatively with ketone bodies and liver triglycerides and with mitochondrial protein acetylation; pointing towards a relation between acetylation, mitochondrial function and fatty acid catabolism in bovine liver during early lactation and negative energy balance (Fig 7).

Our results are in agreement with previous reports of impaired mitochondrial fatty acid oxidation in cows, mice and human patients with fatty liver and ketosis [14,21,54]; but differ with those by Koliaki *et al*., where an increase in maximum electron transport activity was observed in hepatic biopsies from subjects with non-alcoholic fatty liver (NAFL) when compared to healthy subjects [55]. However, since most of the reported data about fatty liver disease come from humans and mouse models, it is not clear if their conclusions can be extrapolated to ruminants.

In dairy cows during lactation oxaloacetate, an intermediary of the Krebs cycle, is channeled towards glucose synthesis, limiting complete oxidation of acetyl-CoA [10] [15]. The increase in acetyl-CoA levels can lead to ketone body synthesis [15], as well as protein acetylation [56,57]. Thus, nutritional management is extremely important during early lactation, when the cow may experience a shortage of glucogenic precursors because demands cannot be fully met by feed intake [8,10]. Pasture-based and TMR systems present different advantages and caveats [58,59]. From a metabolic/nutritional point of view it is generally accepted that TMR systems contribute to an increase in dry matter intake and contain higher levels of non-fiber carbohydrates than pasture based feeding strategies, therefore might result in higher production of propionate in the rumen [8]. Using the NDS Professional software (from RUM&N and Cornell University Department of Animal Science, Reggio Emilia, Italy) based on the

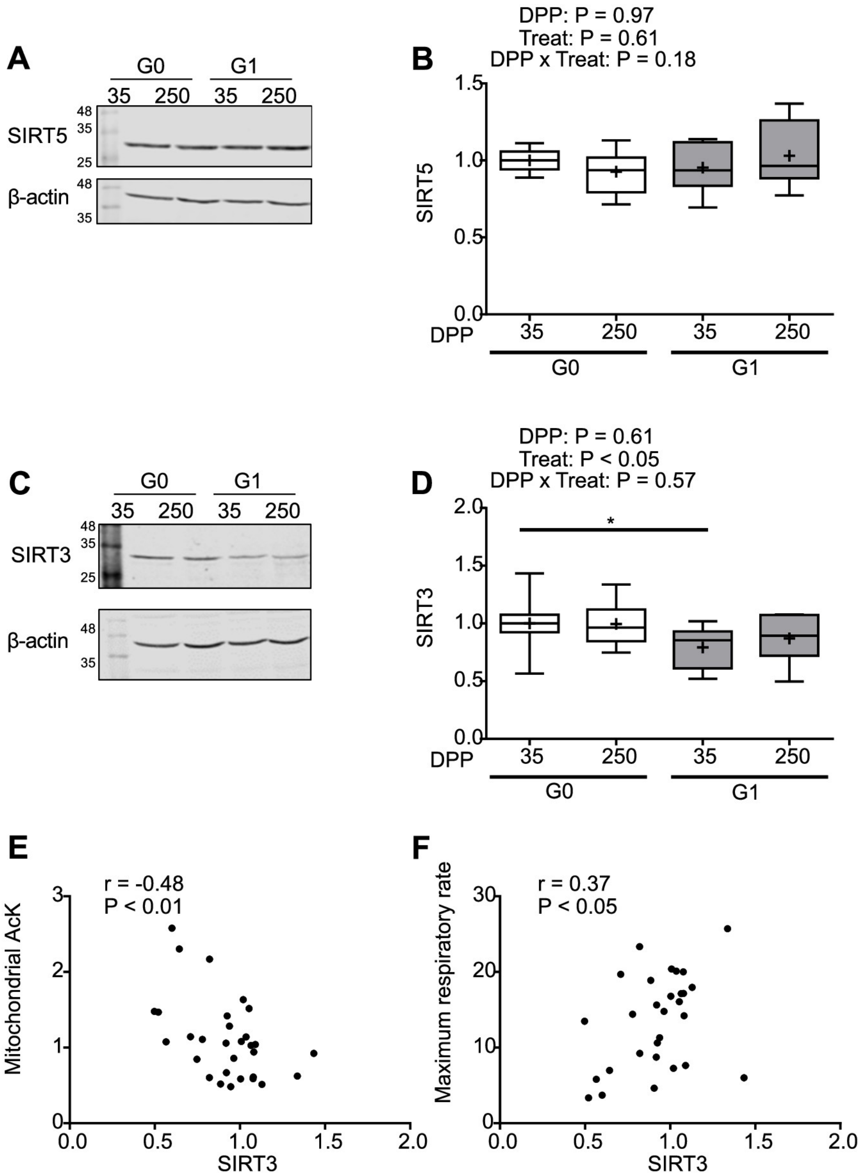

Fig 6. Mitochondrial protein acetylation correlates with a decrease in sirtuin 3 levels. (A and C). Representative western blots of sirtuin 5 (SIRT5) and sirtuin 3 (SIRT3) in liver homogenates from cows of both G0 and G1 groups at 35 and 250 DPP, β-actin was used as loading control. **(B and D)** Independent western blots of sirtuin 5 and sirtuin 3 were quantified by densitometry, normalized with the loading control and expressed in relation to the average value of the G0 group at 35 DPP. **(E)** Shows the correlation between mitochondrial AcK levels and sirtuin 3. **(F)** Shows the correlation between mitochondrial maximum respiratory rate and sirtuin 3. In box plots the box extends from the 25th to 75th percentile, the line in the middle of the box is the median, the cross is the mean and the whiskers represent the minimum and maximum values (N = 8). * $P < 0.05$. G0: Cows were fed TMR *ad libitum* from calving to 180 DPP. G1: Cows grazed *Festuca arundinacea* plus a commercial concentrate or *Medicago* supplemented with TMR (50% of G0 offer), depending on heat stress conditions, from calving to 180 DPP. From 180 to 250 DPP both groups grazed *Medicago sativa* and were supplemented with TMR (50% of G0 offer at 180 DPP).

https://doi.org/10.1371/journal.pone.0213780.g006

model developed by Noziere et al., 2011 [60] we estimated the proportion of the different volatile fatty acids formed in the rumen with the different feeding strategies. These estimations suggested that in the pasture-based system (G1) the acetate:propionate ratio and the non-glucogenic:glucogenic ratio could be higher than in the TMR-system (G0) (2.80 vs. 2.04 and 3.50 vs. 2.65, respectively).

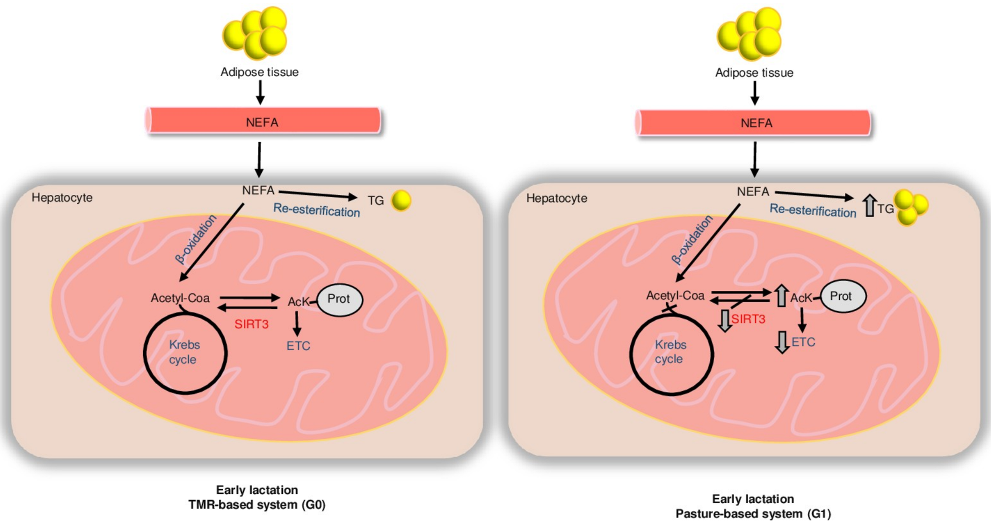

Fig 7. Plausible mechanism behind metabolic changes during early lactation. Lipid reserves are mobilized during early lactation, NEFA reach the blood stream and enter the hepatocyte where they are oxidized to acetyl-CoA in the β-oxidation pathway. The increase in Acetyl-CoA levels leads to acetylation of protein lysine residues. Lower levels of sirtuin 3 (SIRT3) in the liver of G1 cows may contribute to the increase in protein acetylation (AcK-Prot) in the G1 group with respect to the G0 group. Protein lysine acetylation impacts negatively on electron transport chain activity (ETC), Krebs cycle and β-oxidation resulting in an impaired oxidation of NEFA that are re-esterified to triglycerides (TG), giving rise to fatty liver.

https://doi.org/10.1371/journal.pone.0213780.g007

An increase in the supply of propionate might impact positively in oxaloacetate levels, favoring not only gluconeogenesis but also acetyl-CoA oxidation to CO_2 [10]. In agreement with this possibility mitochondrial acetylation was lower in TMR fed cows than in pasture fed cows in early lactation (Fig 7). Additionally, we observed ≈ 70% increase in acetylated protein lysine in isolated mitochondria from pasture fed cows during early lactation, compared to late lactation. Acetylation is a reversible covalent post-translation modification that can both inhibit or increase the activity and stability of key enzymes in β-oxidation, Krebs cycle, ketone body metabolism, electron transport chain and oxidative phosphorylation [49,50,61–63]. Increase in lysine acetylation occurs due to imbalances in acetylation and deacetylation reactions. Mitochondrial acetylation can occur non-enzymatically [56] or catalyzed by acetyltransferases (i.e. acetylase GCN5L1) [64]; while mitochondrial deacetylation is catalyzed by sirtuins 3 and 5 [65].

Previous studies in mice models have shown that sirtuin 3 regulates hepatic fatty acid metabolism [61]. Decreased sirtuin 3 activity and increased acetylation has been observed in mice that develop fatty liver under a high fat diet [20]. On the contrary, during fasting, increased sirtuin 3 levels promote the deacetylation of long-chain acyl coenzyme A dehydrogenase (LCAD) in the liver; which increases enzyme activity and fatty acid oxidation and prevents the accumulation of triglycerides [61]. Thus, different levels of sirtuin 3 could account, at least in part, for the differences in acetylation profiles between G0 and G1 cows during early lactation (Fig 7). Although the contribution to increased acetylation of other events, such as enzymatic or non-enzymatic acetylation cannot be discarded [56,66]. Since sirtuin 3 levels were higher in TMR-fed cows than pasture-fed cows, fatty acid oxidation rates might be higher in the former resulting in less accumulation of liver triglycerides. Our study suggests that higher sirtuin 3 levels in G0 dairy cows during early lactation might be responsible for a better adaptation to excessive energy requirements and that both acetylation and deacetylation reactions can be affected by diet.

Sirtuin 3 is considered a regulator of energy metabolism and homeostasis [20,65,67]. In particular, respiratory chain complexes I-V can be inhibited by acetylation [20,52,68,69] and sirtuin 3 catalyzes their deacetylation increasing electron transport flux and oxidative phosphorylation. Glutamate dehydrogenase is also a sirtuin 3 substrate [70]. Furthermore, Kendrick *et al*. observed that sirtuin 3 knockout results in decreased activity of mitochondrial respiratory complexes III and IV in mice under a high fat diet [20]. Thus, changes in sirtuin 3 and acetylation could underlie the decay in mitochondrial respiration observed during early lactation in pasture-fed cows. Further research is necessary to identify the molecular events behind the diet dependent differences in sirtuin 3 levels.

In human patients and mouse models oxidative stress markers, lipid peroxidation products [71–73] and protein 3-nitrotyrosine [74], correlate with the severity of liver damage and oxidative damage is considered the "second hit" required for the development of inflammation and cytotoxicity [19]. In ruminants, an increase in markers of oxidative stress has been reported in plasma of dairy cows in early lactation [75] and in hepatic biopsies of dairy cows with liver failure during the same period [76]. Nevertheless, the correlation between oxidative stress and liver damage has not yet been elucidated in dairy cows during lactation.

Herein we observed an increase in non-mitochondrial oxygen consumption in early lactation in both G0 and G1 cows compared to late lactation that could be due to an increase in ROS formation (e.g. superoxide or hydrogen peroxide formation by oxidases [27,45,46], or oxygen consumption in lipoperoxidation reactions [47]). Previous studies have shown that lactating cows have higher rates of peroxisomal fatty acid oxidation than non-lactating cows [1,77]. Peroxisomal fatty acid oxidases use oxygen as electron acceptor, reducing it to hydrogen peroxide, and could be responsible for the increase in non-mitochondrial oxygen consumption.

We did not find evidence of an increase in oxidative markers (i.e. 4-HNE-protein adducts or protein 3-nitrotyrosine) in liver homogenates or mitochondria from dairy cows during early lactation, in spite of evidence of triglyceride accumulation in the liver. However, when correlation analyses were performed we found that the cows with the highest 4-HNE-protein adduct levels also had the highest non-mitochondrial oxygen consumption rates, and a positive and significant correlation between these two parameters was obtained. These observations suggest that non-mitochondrial oxygen consumption and lipid peroxidation events might be related. Additionally, no significant correlation could be found between 4-HNE-protein adducts and the decrease in mitochondrial respiration. The fact that fatty liver was not observed in late lactation, that aspartate aminotransferase activity (marker of liver damage) was within normal ranges at all times for practically all the animals and that mitochondrial function was recovered in late lactation suggests that our cows might have not experienced relevant oxidative stress.

Although we cannot dismiss oxidative stress as a potential mediator of mitochondrial impairment, since oxidative damage is challenging to assess *in vivo* [48,78], the changes in mitochondrial function and fatty acid metabolism appear to be due to regulatory events, such as acetylation/deacetylation reactions, rather than irreversible oxidative damage.

Conclusion

In this work we detected changes in respiratory parameters between early and late lactation and found an association between mitochondrial protein acetylation, respiration and fatty acid metabolism in dairy cows in early lactation. Our results show that cows in a pasture-based system, present impaired mitochondrial function during early lactation, increased acetylation of

mitochondrial proteins and decreased levels of sirtuin 3. Overall our results highlight the relevance of nutritional management in this crucial period.

During early lactation, an increase in acetyl-CoA, can promote acetylation of mitochondrial proteins. Higher levels of sirtuin 3 in cows in the TMR-based system versus pasture-based system can counter the increase in acetylation and help maintain mitochondrial homeostasis. However, lower sirtuin 3 levels in cows in the pasture-based system could result in increased acetylation of mitochondrial proteins affecting respiration, oxidative phosphorylation and fatty acid oxidation; potentially leading to accumulation of triglycerides in the liver.

Supporting information

S1 Fig. Subcellular fractionation of liver homogenates. Liver biopsies were homogenized and subcellular fractions enriched in mitochondria, nuclei and cytosol were obtained as described previously [33]. Proteins from the different fractions were resolved by SDS/PAGE and Western blots performed with antibodies against proteins from mitochondria (SDHA), cytosol (β-actin) and nuclei (histone H3).
(TIF)

S2 Fig. Protein tyrosine nitration in liver homogenates. (A) Representative western blot of liver homogenates exposed to different concentrations of peroxynitrite ($ONOO^-$) in 100 mM phosphate buffer pH 7.4. **(B)** Representative western blot of 3-nitrotyrosine in liver homogenates of G0 and G1 cows at 35 and 250 DPP, and a positive control (C (+)). The positive control was obtained exposing the bovine serum albumin to 300 μM peroxynitrite in 100 mM phosphate buffer pH 7.4.
(TIF)

S3 Fig. Evaluation of mitochondrial content in liver biopsies of dairy cows. (A and C) Representative western blots of ATP synthase subunit α (ATP5A) and succinate dehydrogenase subunit A (SDHA) in liver homogenates of cows from both G0 and G1 groups at 35 and 250 DPP. β-actin and tubulin were used as loading controls. **(B and D)** Quantification by densitometry of ATP5A and SDHA levels normalized with the respective loading controls and expressed in relation to the average value of the G0 group at 35 DPP. **(E)** Citrate synthase specific activity was determined in liver homogenates of cows from the G0 and G1 group at 35 and 250 DPP. In box plots the box extends from the 25th to 75th percentile, the line in the middle of the box is the median, the cross is the mean and the whiskers represent the minimum and maximum values (N = 8–10).
(TIF)

Acknowledgments

We would like to thank Daniel Talmón for his help with the estimation of volatile fatty acid formation in the rumen, and to the staff from the Experimental Station "Dr. Mario A. Cassinoni" (EEMAC) for their support in animal handling and Dr. Rafael Radi (CEINBIO, Departamento de Bioquímica, Facultad de Medicina, Universidad de la República, Uruguay) for the anti 3-nitrotyrosine antibody.

Author Contributions

Conceptualization: Mariana Carriquiry, Adriana Cassina, Celia Quijano.

Formal analysis: Mercedes García-Roche, Mariana Carriquiry.

Funding acquisition: Mariana Carriquiry, Adriana Cassina, Celia Quijano.

Investigation: Mercedes García-Roche, Alberto Casal, Mateo Ceriani, Alejandra Jasinsky.

Methodology: Mercedes García-Roche, Diego A. Mattiauda, Mauricio Mastrogiovanni, Andrés Trostchansky, Mariana Carriquiry, Adriana Cassina, Celia Quijano.

Supervision: Mariana Carriquiry, Adriana Cassina, Celia Quijano.

Visualization: Mercedes García-Roche.

Writing – original draft: Mercedes García-Roche, Celia Quijano.

Writing – review & editing: Mariana Carriquiry, Adriana Cassina, Celia Quijano.

References

1. Drackley JK. Biology of Dairy Cows During the Transition Period: the Final Frontier? J Dairy Sci. Elsevier; 1999; 82: 2259–2273. https://doi.org/10.3168/jds.S0022-0302(99)75474-3
2. Wales WJ, Marett LC, Greenwood JS, Wright MM, Thornhill JB, Jacobs JL, et al. Use of partial mixed rations in pasture-based dairying in temperate regions of Australia. Anim Prod Sci. 2013; 53: 1167–1178. https://doi.org/10.1071/AN13207
3. Chilibroste P, Gibb MJ, Soca P, Mattiauda DA. Behavioural adaptation of grazing dairy cows to changes in feeding management: Do they follow a predictable pattern? Anim Prod Sci. 2015; 55: 328–338. https://doi.org/10.1071/AN14484
4. Chilibroste P.; Gibb M.J.; Tamminga S. Pasture Characteristics and Animal Performance. In: Dijkstra J, Forbes J., France J, editors. Quantitative Aspects of Ruminant Digestion and Metabolism. 2nd ed. Wageningen; 2005. pp. 681–706.
5. Meikle A, Adrien M de L, Mattiauda DA, Chilibroste P. Effect of sward condition on metabolic endocrinology during the early postpartum period in primiparous grazing dairy cows and its association with productive and reproductive performance. Anim Feed Sci Technol. Elsevier B.V.; 2013; 186: 139–147. https://doi.org/10.1016/j.anifeedsci.2013.10.003
6. Astessiano A, Carriquiry M, Mattiauda D, Adrien M, Chilibroste P, Meikle A. Endometrial gene expression in primiparous dairy cows at the end of the voluntary waiting period is affected by nutrition: Total mixed ration vs increasing levels of herbage allowance. Reprod Domest Anim. 2017; 1: 1–8. https://doi.org/10.1111/rda.12981 PMID: 28406532
7. Kolver ES, Muller LD. Performance and Nutrient Intake of High Producing Holstein Cows Consuming Pasture or a Total Mixed Ration. J Dairy Sci. Elsevier; 1998; 81: 1403–1411. https://doi.org/10.3168/jds.S0022-0302(98)75704-2
8. Roche JR, Bell AW, Overton TR, Loor JJ. Nutritional management of the transition cow in the 21st century-a paradigm shift in thinking. Anim Prod Sci. 2013; 53: 1000–1023. https://doi.org/10.1071/AN12293
9. Bell AW, Bauman DE. Adaptations of glucose metabolism during pregnancy and lactation. J Mammary Gland Biol Neoplasia. 1997; 2: 265–278. https://doi.org/10.1023/A:1026336505343
10. Aschenbach JR, Kristensen NB, Donkin SS, Hammon HM, Penner GB. Gluconeogenesis in dairy cows: The secret of making sweet milk from sour dough. IUBMB Life. 2010; 62: 869–877. https://doi.org/10.1002/iub.400 PMID: 21171012
11. Ingvartsen KL, Moyes K. Nutrition, immune function and health of dairy cattle. Animal. 2013; 7: 112–122. https://doi.org/10.1017/S175173111200170X
12. Bauman DE, Currie WB. Partitioning of Nutrients During Pregnancy and Lactation: A Review of Mechanisms Involving Homeostasis and Homeorhesis. J Dairy Sci. 1980; 63: 1514–1529. https://doi.org/10.3168/jds.S0022-0302(80)83111-0
13. Baumgard LH, Collier RJ, Bauman DE. A 100-Year Review: Regulation of nutrient partitioning to support lactation. J Dairy Sci. American Dairy Science Association; 2017; 100: 10353–10366. https://doi.org/10.3168/jds.2017-13242 PMID: 29153169
14. Pessayre D, Fromenty B. NASH: A mitochondrial disease. J Hepatol. 2005; 42: 928–940. https://doi.org/10.1016/j.jhep.2005.03.004 PMID: 15885365
15. White HM. The role of TCA cycle anaplerosis in ketosis and fatty liver in periparturient dairy cows. Animals. 2015; 5: 793–802. https://doi.org/10.3390/ani5030384

16. Bobe G, Young JW, Beitz DC. Invited Review: Pathology, Etiology, Prevention, and Treatment of Fatty Liver in Dairy Cows *. J Dairy Sci. Elsevier; 2004; 87: 3105–3124. https://doi.org/10.3168/jds.S0022-0302(04)73446-3

17. Wei Y, Rector RS, Thyfault JP, Ibdah JA. Nonalcoholic fatty liver disease and mitochondrial dysfunction. World J Gastroenterol. 2008; 14: 193–199. https://doi.org/10.3748/wjg.14.193

18. Nassir F, Ibdah J. Role of Mitochondria in Nonalcoholic Fatty Liver Disease. Int J Mol Sci. 2014; 15: 8713–8742. https://doi.org/10.3390/ijms15058713 PMID: 24837835

19. Day CP, James OFW. Steatohepatitis: A tale of two "Hits"? Gastroenterology. 1998; 114: 842–845. https://doi.org/10.1016/S0016-5085(98)70599-2

20. Kendrick AA, Choudhury M, Rahman SM, McCurdy CE, Friederich M, Van Hove JLK, et al. Fatty liver is associated with reduced SIRT3 activity and mitochondrial protein hyperacetylation. Biochem J. 2011; 433: 505–14. https://doi.org/10.1042/BJ20100791

21. Rector RS, Thyfault JP, Uptergrove GM, Morris EM, Naples P, Borengasser SJ, et al. Mitochondrial dysfunction precedes insulin resistance and hepatic steatosis and contributes to the natural history of non- alcoholic fatty liver disease in an obese rodent model. J Hepatol. 2010; 52: 727–736.

22. Quijano C, Trujillo M, Castro L, Trostchansky A. Interplay between oxidant species and energy metabolism. Redox Biol. Elsevier; 2016; 8: 28–42. https://doi.org/10.1016/j.redox.2015.11.010

23. McArt JAA, Nydam D V., Oetzel GR, Overton TR, Ospina PA. Elevated non-esterified fatty acids and β-hydroxybutyrate and their association with transition dairy cow performance [Internet]. Veterinary Journal. Elsevier Ltd; 2013. https://doi.org/10.1016/j.tvjl.2013.08.011

24. Han van der Kolk JH, Gross JJ, Gerber V, Bruckmaier RM. Disturbed bovine mitochondrial lipid metabolism: a review. Vet Q. Taylor & Francis; 2017; 37: 262–273. https://doi.org/10.1080/01652176.2017.1354561 PMID: 28712316

25. Mizutani H, Sako T, Toyoda Y, Kawabata T, Urumuhang N, Koyama H, et al. Preliminary Studies on Hepatic Carnitine Palmitoyltransferase in Dairy Cattle with or without Fatty Liver. Vet Res Commun. 1999; 23: 475–480. https://doi.org/10.1023/a:1006358222037 PMID: 10672964

26. Gao W, Du X, Lei L, Wang H, Zhang M, Wang Z, et al. NEFA-induced ROS impaired insulin signalling through the JNK and p38MAPK pathways in non-alcoholic steatohepatitis. J Cell Mol Med. 2018; 1: 1–15. https://doi.org/10.1111/jcmm.13617

27. Brand MD, Nicholls DG. Assessing mitochondrial dysfunction in cells. 2011; 312: 297–312. https://doi.org/10.1042/BJ20110162 PMID: 21726199

28. NRC Nutrient Requirements of Dairy Cattle. Washington National Academies Press; 2001.

29. Johnson HD, K H.H., R A.C., B I.L, Shanklin. Role of heat tolerance and production level in response of lactating Holsteins to various temperature-humidity conditions. J Dairy Sci. 1961; 44.

30. Edmonson AJ, Lean IJ, Weaver LD, Farver T, Webster G. A Body Condition Scoring Chart for Holstein Dairy Cows. J Dairy Sci. Elsevier; 1989; 72: 68–78. https://doi.org/10.3168/jds.S0022-0302(89)79081-0

31. Carriquiry M, Weber WJ, Fahrenkrug SC, Crooker BA. Hepatic gene expression in multiparous Holstein cows treated with bovine somatotropin and fed n-3 fatty acids in early lactation. J Dairy Sci. Elsevier; 2009; 92: 4889–4900. https://doi.org/10.3168/jds.2008-1676

32. García-Roche M, Casal A, Carriquiry M, Radi R, Quijano C, Cassina A. Respiratory analysis of coupled mitochondria in cryopreserved liver biopsies. Redox Biol. Elsevier B.V.; 2018; 17: 207–212. https://doi.org/10.1016/j.redox.2018.03.008 PMID: 29704825

33. Dimauro I, Pearson T, Caporossi D, Jackson MJ. A simple protocol for the subcellular fractionation of skeletal muscle cells and tissue. BMC Res Notes. BMC Research Notes; 2012; 5: 1.

34. Pesta D, Gnaiger E. High-Resolution Respirometry: OXPHOS Protocols for Human Cells and Permeabilized Fibers from Small Biopsies of Human Muscle. In: Palmeira CM, Moreno AJ, editors. Mitochondrial Bioenergetics: Methods and Protocols, Methods in Molecular Biology. New York: Springer; 2012. pp. 25–58. https://doi.org/10.1007/978-1-61779-382-0_3

35. Spinazzi M, Casarin A, Pertegato V, Salviati L, Angelini C. Assessment of mitochondrial respiratory chain enzymatic activities on tissues and cultured cells. Nat Protoc. 2012; 7: 1235–1246. https://doi.org/10.1038/nprot.2012.058

36. Bradford MM. A rapid and sensitive method for the quantitation of microgram quantities of protein utilizing the principle of protein-dye binding. Anal Biochem. 1976; 72: 248–254. https://doi.org/10.1016/0003-2697(76)90527-3

37. Hara A, Radin NS. Lipid extraction of tissues with a low toxicity solvent. Anal Biochem. 1978; 90: 420–426. https://doi.org/10.1016/0003-2697(78)90046-5

38. Trostchansky A, Souza M, Ferreira A, Ferrari M, Blanco F, Trujillo M, et al. Synthesis, Isomer Characterization, and Anti-Inflammatory Properties of Nitroarachidonate. Biochemistry. 2007; 46: 4645–4653. https://doi.org/10.1021/bi602652j

39. Porter WH, Yao HH, Karounos DG. Laboratory and clinical evaluation of assays for beta-hydroxybutyrate. Am J Clin Pathol. 1997; 107: 353–358.

40. Chilliard Y, Bauchart D, Barnouin J, Duboisset F, Flechet J, Charcornac JP. Determination of plasma non-esterified fatty acids in herbivores and man: a comparison of values obtained by manual or automatic chromatographic, titrimetric, colorimetric and enzymatic methods. Reprod Nutr Dev. 1984; 24: 469–482. https://doi.org/10.1051/rnd:19840412

41. Schumann G, Bonora R, Ceriotti F, Férard G, Ferrero C, Franck P, et al. IFCC primary reference procedures for the measurement of catalytic activity concentrations of enzymes at 37 degrees C. International Federation of Clinical Chemistry and Laboratory Medicine. Part 5. Reference procedure for the measurement of catalytic con. Clin Chem Lab Med. 2002; 40: 725–733. https://doi.org/10.1515/CCLM.2002.125

42. Kirovski D, Sladojevic Z. Prediction and Diagnosis of Fatty Liver in Dairy Cows. SM J Gastroenterol Hepatol. 2017; 3: 1–7.

43. Herdt TH. Fatty liver in dairy cows. Vet Clin North Am Food Anim Pr. 1988; 4: 269–287. https://doi.org/10.1016/S0749-0720(15)31048-3

44. Rolo AP, Teodoro JS, Palmeira CM. Role of oxidative stress in the pathogenesis of nonalcoholic steatohepatitis. Free Radic Biol Med. Elsevier Inc.; 2012; 52: 59–69. https://doi.org/10.1016/j.freeradbiomed.2011.10.003 PMID: 22064361

45. Kramer PA, Chacko BK, Ravi S, Johnson MS, Mitchell T, Darley-Usmar VM. Bioenergetics and the Oxidative Burst: Protocols for the Isolation and Evaluation of Human Leukocytes and Platelets. J Vis Exp. 2014; 85: 1–9. https://doi.org/10.3791/51301

46. Chacko BK, Kramer PA, Ravi S, Benavides GA, Mitchell T, Dranka BP, et al. The Bioenergetic Health Index: a new concept in mitochondrial translational research. Clin Sci. 2014; 127: 367–373. https://doi.org/10.1042/CS20140101 PMID: 24895057

47. Schaur RJ, Siems W, Bresgen N, Eckl PM. 4-Hydroxy-Nonenal—a Bioactive Lipid Peroxidation Product. Biomolecules. 2015. https://doi.org/10.3390/biom5042247

48. Radi R, Peluffo G, Alvarez MN, Naviliat M, Cayota A. Unraveling peroxynitrite formation in biological systems. Free Radic Biol Med. 2001; 30: 463–488. https://doi.org/10.1016/S0891-5849(00)00373-7 PMID: 11182518

49. Anderson KA, Hirschey MD. Mitochondrial protein acetylation regulates metabolism. Essays Biochem. 2012; 52: 23–35. https://doi.org/10.1042/bse0520023 PMID: 22708561

50. Wagner GR, Payne RM. Mitochondrial Acetylation and Diseases of Aging. J Aging Res. 2011; 2011: 1–13. https://doi.org/10.4061/2011/234875

51. Choudhary C, Kumar C, Gnad F, Nielsen ML, Rehman M, Walther TC, et al. Lysine Acetylation Targets Protein Complexes and Co-Regulates Major Cellular Functions. Science (80-). 2009; 325: 834–840. https://doi.org/10.1126/science.1175371

52. Ahn B-H, Kim H-S, Song S, Lee IH, Liu J, Vassilopoulos A, et al. A role for the mitochondrial deacetylase Sirt3 in regulating energy homeostasis. Proc Natl Acad Sci. 2008; 105: 14447–14452. https://doi.org/10.1073/pnas.0803790105 PMID: 18794531

53. Verdin E, Hirschey MD, Finley LWS, Haigis MC. Sirtuin regulation of mitochondria: Energy production, apoptosis, and signaling. Trends Biochem Sci. Elsevier Ltd; 2010; 35: 669–675. https://doi.org/10.1016/j.tibs.2010.07.003

54. Thyfault JP, Rector RS, Uptergrove GM, Borengasser SJ, Morris EM, Wei Y, et al. Rats selectively bred for low aerobic capacity have reduced hepatic mitochondrial oxidative capacity and susceptibility to hepatic steatosis and injury. J Physiol. 2009; 587: 1805–1816. https://doi.org/10.1113/jphysiol.2009.169060

55. Koliaki C, Szendroedi J, Kaul K, Jelenik T, Nowotny P, Jankowiak F, et al. Adaptation of Hepatic Mitochondrial Function in Humans with Non-Alcoholic Fatty Liver Is Lost in Steatohepatitis. Cell Metab. Elsevier Inc.; 2015; 21: 739–746. https://doi.org/10.1016/j.cmet.2015.04.004 PMID: 25955209

56. Wagner GR, Payne RM. Widespread and Enzyme-independent N ♪ -Acetylation and N ♪ -Succinylation of Proteins in the Chemical Conditions of the Mitochondrial Matrix *. J Biol Chem. 2013; 288: 29036–29045. https://doi.org/10.1074/jbc.M113.486753

57. Pougovkina O, Te Brinke H, Ofman R, Van Cruchten AG, Kulik W, Wanders RJA, et al. Mitochondrial protein acetylation is driven by acetyl-CoA from fatty acid oxidation. Hum Mol Genet. 2014; 23: 3513–3522. https://doi.org/10.1093/hmg/ddu059 PMID: 24516071

58. Hills JL, Wales WJ, Dunshea FR, Garcia SC, Roche JR. Invited review: An evaluation of the likely effects of individualized feeding of concentrate supplements to pasture-based dairy cows. J Dairy Sci. Elsevier; 2015; 98: 1363–1401. https://doi.org/10.3168/jds.2014-8475 PMID: 25582585
59. Kolver ES, Roche JR, De Veth MJ, Thorne PL, Napper AR. Total mixed ratios versus pasture diets. Evidence for a genotype x diet interaction in dairy cow performance. Proc New Zeal Soc An. 2002; 62: 246–251.
60. Nozière P, Glasser F, Sauvant D. In vivo production and molar percentages of volatile fatty acids in the rumen: A quantitative review by an empirical approach. Animal. 2011; 5: 403–414. https://doi.org/10.1017/S1751731110002016
61. Hirschey MD, Shimazu T, Goetzman E, Jing E, Schwer B, Lombard DB, et al. SIRT3 regulates mitochondrial fatty-acid oxidation by reversible enzyme deacetylation. Nature. 2010; 464: 121–125. https://doi.org/10.1038/nature08778 PMID: 20203611
62. Wang Q, Zhang Y, Yang C, Xiong H, Lin Y, Yao J, et al. Acetylation of Metabolic Enzymes Coordinates Carbon Source Utilization and Metabolic Flux. Science (80-). 2010; 327: 1004–1007. https://doi.org/10.1126/science.1179687
63. Alrob OA, Sankaralingam S, Ma C, Wagg CS, Fillmore N, Jaswal JS, et al. Obesity-induced lysine acetylation increases cardiac fatty acid oxidation and impairs insulin signalling. Cardiovasc Res. 2014; 103: 485–497. https://doi.org/10.1093/cvr/cvu156
64. Drazic A, Myklebust LM, Ree R, Arnesen T. The world of protein acetylation. Biochim Biophys Acta. The Authors; 2016; 1864: 1372–1401. https://doi.org/10.1016/j.bbapap.2016.06.007 PMID: 27296530
65. Osborne B, Cooney GJ, Turner N. Are sirtuin deacylase enzymes important modulators of mitochondrial energy metabolism? Biochim Biophys Acta—Gen Subj. Elsevier B.V.; 2014; 1840: 1295–1302. https://doi.org/10.1016/j.bbagen.2013.08.016 PMID: 23994496
66. Thapa D, Zhang M, Manning JR, Guimarães DA, Stoner MW, O'Doherty RM, et al. Acetylation of mitochondrial proteins by GCN5L1 promotes enhanced fatty acid oxidation in the heart. Am J Physiol—Hear Circ Physiol. 2017; 313: H265–H274. https://doi.org/10.1152/ajpheart.00752.2016 PMID: 28526709
67. Newman JC, He W, Verdin E. Mitochondrial protein acylation and intermediary metabolism: Regulation by sirtuins and implications for metabolic disease. J Biol Chem. 2012; 287: 42436–42443. https://doi.org/10.1074/jbc.R112.404863
68. Wu YT, Lee HC, Liao CC, Wei YH. Regulation of mitochondrial FoF1ATPase activity by Sirt3-catalyzed deacetylation and its deficiency in human cells harboring 4977bp deletion of mitochondrial DNA. Biochim Biophys Acta—Mol Basis Dis. Elsevier B.V.; 2013; 1832: 216–227. https://doi.org/10.1016/j.bbadis.2012.10.002 PMID: 23046812
69. Finley LWS, Haas W, Desquiret-Dumas V, Wallace DC, Procaccio V, Gygi SP, et al. Succinate dehydrogenase is a direct target of sirtuin 3 deacetylase activity. PLoS One. 2011; 6: 4–9. https://doi.org/10.1371/journal.pone.0023295 PMID: 21858060
70. Lombard DB, Alt FW, Cheng H-L, Bunkenborg J, Streeper RS, Mostoslavsky R, et al. Mammalian Sir2 Homolog SIRT3 Regulates Global Mitochondrial Lysine Acetylation. Mol Cell Biol. 2007; 27: 8807–8814. https://doi.org/10.1128/MCB.01636-07 PMID: 17923681
71. Yesilova Z, Yaman H, Oktenli C, Ozcan A, Uygun A, Cakir E, et al. Systemic markers of lipid peroxidation and antioxidants in patients with nonalcoholic fatty liver disease. Am J Gastroenterol. 2005; 100: 850–855. https://doi.org/10.1111/j.1572-0241.2005.41500.x
72. Seki S, Kitada T, Yamada T, Sakaguchi H, Nakatani K, Wakasa K. In situ detection of lipid peroxidation and oxidative DNA damage in non-alcoholic fatty liver diseases. J Hepatol. 2002; 37: 56–62. https://doi.org/10.1016/S0168-8278(02)00073-9
73. Spahis S, Delvin E, Borys J-M, Levy E. Oxidative Stress as a Critical Factor in Nonalcoholic Fatty Liver Disease Pathogenesis. Antioxid Redox Signal. 2017; 26: 519–541. https://doi.org/10.1089/ars.2016.6776 PMID: 27452109
74. Sanyal AJ, Campbell-Sargent C, Mirshahi F, Rizzo WB, Contos MJ, Sterling RK, et al. Nonalcoholic steatohepatitis: Association of insulin resistance and mitochondrial abnormalities. Gastroenterology. American Gastroenterological Association; 2001; 120: 1183–1192. https://doi.org/10.1053/gast.2001.23256
75. Castillo C, Hernandez J, Bravo A, Lopez-Alonso M, Pereira V, Benedito JL. Oxidative status during late pregnancy and early lactation in dairy cows. Vet J. 2005; 169: 286–292. https://doi.org/10.1016/j.tvjl.2004.02.001
76. Mudron P, Rehage J, Qualmann K, Sallmann H-P, Scholz H. A Study of Lipid Peroxidation and Vitamin E in Dairy Cows with Hepatic Insufficiency. J Vet Med Ser A. 1999; 46: 219–224. https://doi.org/10.1046/j.1439-0442.1999.00206.x

77. Grum DE, Hansen LR, Drackley JK. Peroxisomal beta-oxidation of fatty acids in bovine and rat liver. Comp Biochem Physiol B Biochem Mol Biol. 1994; 109: 281–292. https://doi.org/10.1016/0305-0491(94)90012-4

78. Halliwell B, Whiteman M. Measuring reactive species and oxidative damage in vivo and in cell culture: how should you do it and what do the results mean? Br J Pharmacol. 2004; 142: 231–255. https://doi.org/10.1038/sj.bjp.0705776
